# Understanding the Role of Borohydride Doping in Electrochemical Stability of Argyrodite Li_6_PS_5_Cl Solid‐State Electrolyte

**DOI:** 10.1002/adma.202506095

**Published:** 2025-07-15

**Authors:** Yixian Wang, Vikalp Raj, Qianqian Yan, Cole D. Fincher, Yuanshun Li, Rohit Raj, Hugo Celio, Andrei Dolocan, Guang Yang, Frédéric A. Perras, Yet‐Ming Chiang, John Watt, Hong Fang, Puru Jena, David Mitlin

**Affiliations:** ^1^ Materials Science and Engineering Program Walker Department of Mechanical Engineering and Texas Materials Institute The University of Texas at Austin Austin TX 78712 USA; ^2^ Department of Materials Science & Engineering Massachusetts Institute of Technology Cambridge MA 02139 USA; ^3^ Chemical Sciences Division Oak Ridge National Laboratory Oak Ridge TN 37830 USA; ^4^ Chemical and Biological Sciences Division Ames National Laboratory Ames IA 50011 USA; ^5^ Department of Chemistry Iowa State University Ames IA 50011 USA; ^6^ Center for Integrated Nanotechnologies Los Alamos National Laboratory Los Alamos NM 87545 USA; ^7^ Department of Physics Rutgers University Camden NJ 08102 USA; ^8^ Center for Computational and Integrative Biology Rutgers University Camden NJ 08103 USA; ^9^ Department of Physics Virginia Commonwealth University Richmond VA 23238 USA

**Keywords:** argyrodite, borohydride, inorganic solid electrolyte, polyanion, solid‐state battery

## Abstract

This work elucidates the mechanism by which lithium borohydride (LiBH_4_) doping into argyrodite‐type Li_6_PS_5_Cl (LBH‐LPSCl) solid‐state electrolyte (SSE) enhances electrochemical stability. State‐of‐the‐art electrochemical performance is achieved with 5 wt% borohydride. Symmetric cells achieve critical current density (CCD) of 7.3 mA cm^−2^, versus 2.6 mA cm^−2^ for baseline‐LPSCl. All solid‐state batteries (ASSBs) employing lithium metal and NMC811 cathode are stable over 400 cycles at 0.5C, with capacity retention of 83%. An anode‐free ASSB (AF‐ASSB) is stable over 600 cycles, with capacity loss of 0.04% per cycle. 5LBH‐LPSCl allows for enhanced low temperature operation, down to −14 °C. Yet the difference in electrolytes’ bulk microstructures and hardnesses are minimal, while ionic conductivity is incrementally improved (≈50%). Theoretical modeling indicates limited effect of substitution on thermodynamic stability of PS_4_^3−^ units, which decompose when contacting Li. Instead, enhanced electrochemical stability is site‐specific kinetic effect: In situ electrodeposition experiments using X‐ray photoelectron spectroscopy (XPS) and time‐of‐flight secondary ion mass spectrometry (TOF‐SIMS) reveal tri‐layer SEI based predominately on Li_3_P/LiBH_4_/Li_2_S that blocks electrons while facilitating ion transport. This SEI manifests reduced interface resistance and accelerated nucleation and growth of metallic Li. With baseline‐LPSCl the SEI based on Li_3_P/Li_2_S is substantially thicker, generating localized stresses that promote interfacial cracking while cycling.

## Graphical abstract

This study reveals how LiBH_4_ doping in argyrodite‐type Li_6_PS_5_Cl solid‐state electrolyte improves electrochemical stability by forming a tri‐layer SEI based predominately on Li_3_P/LiBH_4_/Li_2_S that promotes ion transport and blocks electrons. In situ XPS and TOF‐SIMS show thinner SEI, enhanced Li electrodeposition, and better interfacial wetting compared to baseline LPSCl, highlighting the role of B‐rich layers in interface engineering.

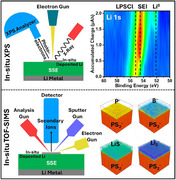

## 1 Introduction

Safety and energy density are critical considerations in the design of next‐generation electrochemical storage devices, particularly for electric vehicles (EVs).^[^ ^1^, ^2^, ^3^ ^]^ All‐solid‐state batteries (ASSBs) have emerged as a viable solution due to the use of inorganic, non‐flammable solid‐state electrolytes (SSEs), which offer greater thermal stability compared to organic‐based liquid electrolytes.^[^ ^4^, ^5^ ^]^ Additionally, the potential compatibility with lithium metal anodes allows for achieving a high specific energy of 500 Wh kg^−1^ at the cell level when paired with high‐voltage cathode materials.^[^ ^6^, ^7^ ^]^ Recent advancements have focused on the development of novel inorganic SSEs with enhanced ionic conductivity.^[^ ^8^, ^9^, ^10^ ^]^ Among these, sulfide SSEs including glassy and glassy‐ceramic binary Li_2_S‐P_2_S_5_,^[^ ^11^, ^12^ ^]^ thio‐LISICON (Li_10_GeP_2_S_12_),^[^ ^13^, ^14^, ^15^ ^]^ argyrodite‐type Li_6_PS_5_X (X = Cl, Br, I)^[^ ^16^, ^17^, ^18^ ^]^ have attracted significant research interest due to their higher ionic conductivity (>1 mS cm^−1^ at room temperature) and mechanical softness, which facilitates improved interfacial contact with Li metal foils and ceramic cathode particles.

Despite these advantages, sulfide‐based SSEs face several critical challenges, particularly their susceptibility to short‐circuit during prolonged cycling due to dendrite growth.^[^ ^19^, ^20^, ^21^ ^]^ Dendrite formation and propagation are widely observed across various SSEs and are attributed to localized, non‐uniform electrodeposition and dissolution of lithium. This process intensifies mechanical stresses within the electrolyte, leading to fractures of SSE and eventual short‐circuiting of the cell.^[^ ^22^, ^23^, ^24^ ^]^ Meanwhile, interface defects such as voids and pre‐existing cracks can act as hot spots, trapping electrons and inducing lithium‐ion reduction.^[^ ^25^, ^26^, ^27^ ^]^ This leads to direct Li deposition and dendrite growth within the SSE. These destructive effects worsen at higher current densities where void accumulation at the Li/SSE interface outpaces lithium replenishment by self‐diffusion. Therefore, the critical current density (CCD), defined as the maximum current density a cell can sustain before short‐circuiting, serves as a key parameter for assessing the performance of SSEs. Factors such as capacity, temperature, and pressure influence the CCD.^[^ ^28^, ^29^, ^30^ ^]^ For example, Bruce *et al.* revealed that the CCD of the Li/LPSCl/Li symmetric cells is strongly correlated with the external pressure applied during cycling.^[^ ^31^ ^]^

Another major limitation of argyrodite SSEs is their reactivity with electrodes, leading to chemo‐mechanical effects stemming from their narrow thermodynamic stability window (typically 1.7‐2.0 V).^[^ ^32^, ^33^, ^34^ ^]^ These electrolytes undergo reductive and oxidative decomposition into equilibrium and/or metastable phases even when in contact with inert blocking electrodes such as copper current collectors.^[^ ^35^, ^36^, ^37^ ^]^ Prolonged interfacial reactions progressively increase internal resistance, causing cell failure due to impedance buildup rather than short‐circuiting. In addition, sulfide SSEs like Li_10_GeP_2_S_12_ (LGPS) and Li_10_SnP_2_S_12_ (LSPS) decompose into a solid electrolyte interphase (SEI) comprising electronically conducting products of Ge/Sn and Li‐Ge/Li‐Sn alloys, in addition to Li_2_S and Li_3_P.^[^ ^38^, ^39^, ^40^ ^]^ These phases exhibit an unfavorable combination of low ionic conductivity and sufficient electronic conductivity, resulting in the formation of a mixed conducting interphase (MCI) that promotes continuous electrolyte decomposition. The reductive decomposition of Li_6_PS_5_Cl (LPSCl) SSE begins at voltages below 1.7 V versus Li/Li^+^, forming an SEI composed of Li_3_P, Li_2_S and LiCl.^[^ ^41^, ^42^, ^43^, ^44^ ^]^ While the presence of LiCl in the SEI provides a certain degree of self‐passivation, the relatively high electrical conductivity of the equilibrium Li_3_P phase contributes to an increase in internal battery resistance, ultimately degrading the electrochemical performance. Hence, constructing a stable Li/SSE interface is essential to improving the performance of ASSBs.

Electrolyte doping has emerged as one of the most effective strategies to stabilize Li/SSE interfaces. Halogen doping (e.g., F, Cl, Br, I) enhances the electrochemical stability of SSEs and promotes the formation of robust SEIs enriched with lithium halides.^[^ ^45^, ^46^, ^47^, ^48^ ^]^ Similarly, oxygen doping improves redox stability, structural integrity, and interface compatibility, thereby mitigating side reactions and dendrite growth.^[^ ^49^, ^50^, ^51^ ^]^ However, as one of the side effects, these dopants often reduce the ionic conductivity of SSEs. In addition to halide dopants, organic lithium salts have also been explored as effective dopants or surface coatings for LPSCl SSEs to improve their chemical stability. For example, Liu *et al.* synthesized LiTFSI@LPSC using mechanical milling, the resulting SSE enabled stable full‐cell performance owing to the formation of fluorinated interphases on both the anode and cathode sides.^[^ ^52^ ^]^ Similarly, Yang *et al.* developed a LiDFOB‐coated LPSCl SSE using a ball‐milling method, which extended the electrochemical window on the cathode side to 4.5 V when paired with a LiCoO_2_ (LCO) cathode.^[^ ^53^ ^]^

Lithium borohydride (LiBH_4_), widely studied for hydrogen storage applications,^[^ ^54^, ^55^, ^56^ ^]^ has drawn interest as a potential electrolyte for SSBs due to its high ionic conductivity (1 mS cm^−1^, high‐temperature hexagonal phase) and good compatibility with lithium metal.^[^ ^57^, ^58^, ^59^ ^]^ Table S1, Supporting Information displays the reaction pathways and reaction energies of LPSCl with various additives, as predicted by Materials Project.^[^ ^60^ ^]^ It may be observed that the unique feature of LPSCl + LiBH_4_ system is that it is chemically unreactive, with no predicted reaction products or decomposition pathways. Additionally, LiBH_4_ demonstrates considerable thermal stability, with no hydrogen release observed up to 330 °C or higher, which is far above the typical operating temperatures of practical SSBs.^[^ ^61^, ^62^, ^63^ ^]^ The BH_4_^−^ doped argyrodite SSEs have demonstrated enhanced ionic conductivity and higher electrochemical stability compared to LPSCl. The theoretical underpinning for this approach was first demonstrated by Fang and Jena.^[^ ^64^, ^65^, ^66^, ^67^ ^]^ The authors employed modeling to demonstrate that replacing halogens (at Cl^−^ sites) with polyanions such as BH_4_^−^ would not only lead to enhanced ionic conductivity but also enhanced stability. The rationale was that the increased electron affinity, as well as the non‐spherical geometry of the BH_4_^−^, changes the potential energy surface as well as introduces new pathways for ion migration. For example, Ceder *et al.* fabricated BH_4_‐substituted Li argyrodite (Li_5.91_PS_4.91_(BH_4_)_1.09_) using a mechanochemical method, achieving an enhanced ionic conductivity of 4.8 mS cm^−1^.^[^ ^68^ ^]^ Cho *et al.* prepared a Li_5.25_PS_4.25_(BH_4_)_1.75_ via a ball‐milling method, reporting a superior ionic conductivity of up to 13.8 mS cm^−1^.^[^ ^69^ ^]^ Additionally, partially substituted Li_6_PS_5_Cl_1−x_(BH_4_)_x_ SSEs have been synthesized, demonstrating improved ionic conductivity and enhanced electrochemical stability.^[^ ^70^, ^71^ ^]^ While the structures of BH_4_‐doped SSEs and their relationships to ionic conductivity are well‐documented both experimentally and theoretically, the mechanisms underlying their stabilizing effects at the Li/SSE interface remain poorly understood.

In this study, we elucidate the role of BH_4_‐doping in enhancing interfacial stability of LiBH_4_‐doped LPSCl SSE (LBH‐LPSCl). Advanced analytical methods such as site‐specific cryogenic focused ion beam (cryo‐FIB) analysis, in situ X‐ray photoelectron spectroscopy (XPS) and time‐of‐flight secondary ion mass spectrometry (TOF‐SIMS) are combined with theoretical modeling. The material is synthesized through a planetary mechanical wet ball milling of commercially purchased argyrodite LPSCl powder and LiBH_4_ powder. The optimized SSE (5LBH‐LPSCl, 5 wt% LiBH_4_) delivers state‐of‐the‐art electrochemical performance without requiring artificial SEI layers or additional secondary modifications to the lithium metal or current collector surfaces. In situ Li electrodeposition analysis reveals the evolution of the SEI and its compositional changes over time. A faster rate of lithium metal growth is observed with 5LBH‐LPSCl compared to the baseline SSE. In the baseline unmodified LPSCl displays a bilayer SEI structure, with a S‐rich layer (predominately Li_2_S) facing metal, and a P‐rich (predominately Li_3_P) facing the parent SSE. With LBH doping, a B‐rich layer (predominately LiBH_4_) exists between these two layers, acting as an additional passivation barrier that enhances interfacial stability. This trilayer SEI is substantially thinner than the baseline SEI, indicating its greater propensity for interface passivation. Theoretical modeling further elucidates the mechanisms by which LiBH_4_ doping affects the LPSCl structure and electrochemical stability.

## 2 Results and Discussion

The LiBH_4_‐doped Li_6_PS_5_Cl (LBH‐LPSCl) solid‐state electrolytes (SSEs) were prepared using a planetary mechanical milling method in a liquid media. Briefly, commercially purchased LPSCl powder was mixed with LiBH_4_ powder at LiBH_4_:LPSCl weight ratios of 1:100, 1:20, and 1:10. The mixtures were subjected to planetary ball milling in m‐xylene solution with zirconium oxide balls as the milling media. The milling process was conducted at 300 rpm, with intermittent 5‐min rest periods after every 20 min of milling to facilitate heat dissipation. Given that LiBH_4_ melts at ≈280 °C and decomposes at around 330 °C,^[^ ^61^, ^62^, ^63^ ^]^ these milling conditions are unlikely to cause electrolyte decomposition due to overheating. Based on our prior optimization studies the total milling time was set to 24 h.^[^ ^72^ ^]^ The resulting loose powders, as well as the SSE pellets formed by compacting these powders, are designated as “xLBH‐LPSCl” (x = 1, 5, or 10), where “x” represents the weight ratio (wt%) of the LBH additive to LPSCl SSE. For comparative analysis, two additional lithium polyanionic salts, LiBF_4_ and LiPF_6_, were incorporated into LPSCl SSEs using the same procedure at a fixed ratio of 1:20. These samples are labeled as “5LBF‐LPSCl” and “5LPF‐LPSCl,” respectively. As a baseline identically planetary ball‐milled LPSCl without any additive was prepared and is referred to as “baseline‐LPSCl.”

**Figure 1:**
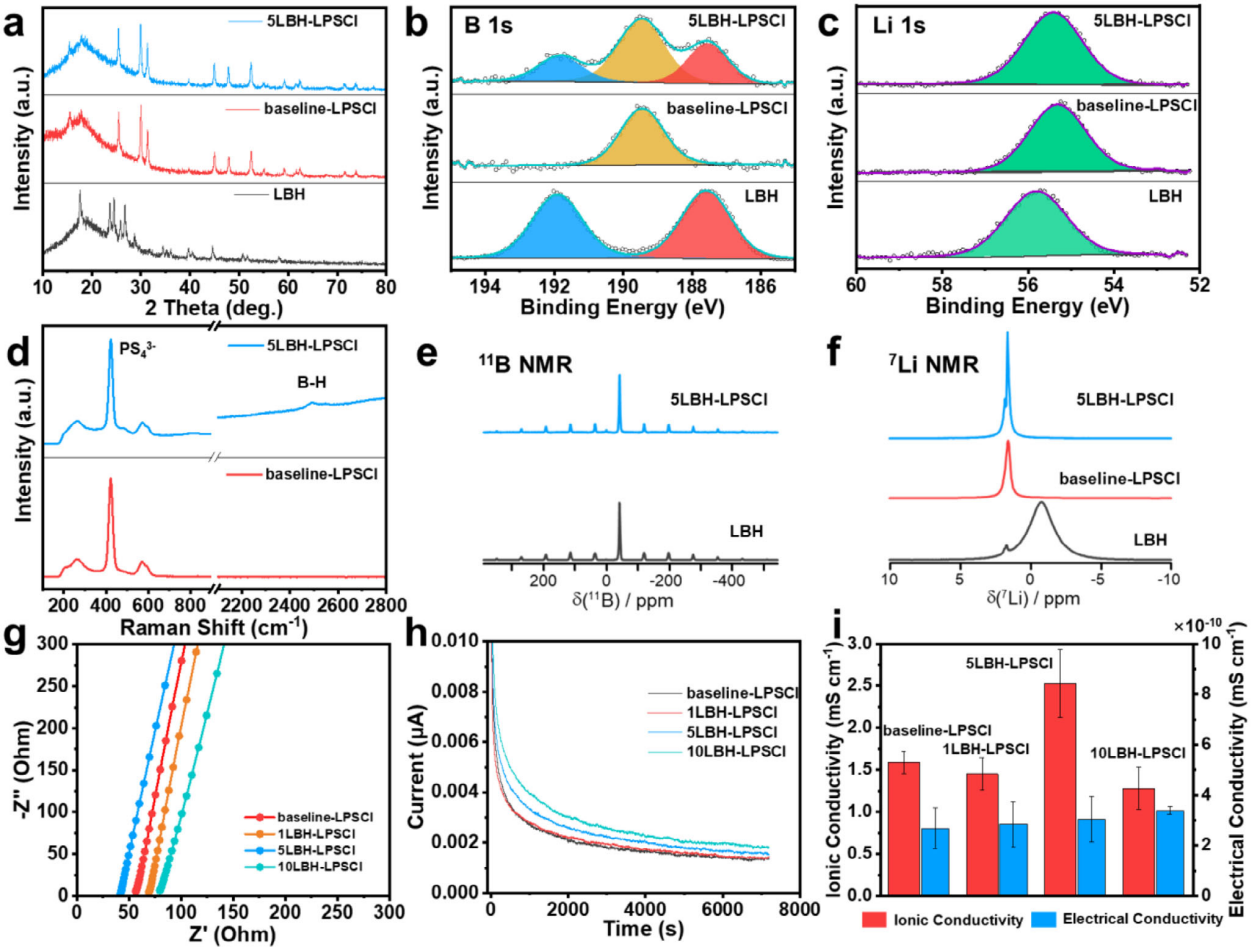
a) XRD profiles, high‐resolution XPS b) B 1s and c) Li 1s spectra, d) Raman spectra, solid‐state NMR e) ^11^B and f) ^7^Li spectra of 5LBH‐LPSCl, baseline‐LPSCl, and as‐received LBH powder samples. g) Nyquist plots and h) current‐time curve under constant voltage hold at 0.5 V of LBH‐doped and baseline‐LPSCl samples. i) Comparison of ionic and electrical conductivities of LBH‐doped and baseline‐LPSCl.a) XRD profiles, high‐resolution XPS b) B 1s and c) Li 1s spectra, d) Raman spectra, solid‐state NMR e) ^11^B and f) ^7^Li spectra of 5LBH‐LPSCl, baseline‐LPSCl, and as‐received LBH powder samples. g) Nyquist plots and h) current‐time curve under constant voltage hold at 0.5 V of LBH‐doped and baseline‐LPSCl samples. i) Comparison of ionic and electrical conductivities of LBH‐doped and baseline‐LPSCl.

**Figure** **1**a presents the X‐ray diffraction (XRD) patterns of 5LBH‐LPSCl, baseline‐LPSCl, and unprocessed as‐received LBH powder. After the mechanical mixing of LPSCl and LBH, no additional peaks were observed in the 5LBH‐LPSCl sample compared to baseline‐LPSCl. Meanwhile all the characteristic crystalline peaks of LBH disappeared entirely. There are no detectable LBH crystallite peaks up to the detection limit of the bench‐top XRD instrument. Furthermore, the relative peak intensity of 5LBH‐LPSCl remains comparable to that of baseline‐LPSCl. This indicates that co‐milling does minimal damage to the LPSCl crystal structure. Figure S1, Supporting Information shows the XRD profiles of LBH‐LPSCl SSEs with varying LBH doping levels. These profiles exhibit minimal differences, with no LBH peaks being detected even at a 10 wt% doping level. In addition to being substituted into the LPSCl structure, it is possible that some of the LBH remains a separate phase but is amorphized. This would likewise eliminate the characteristic crystalline peaks for the material. As will be demonstrated, away from the SEI, the effect of doping on the bulk properties of LPSCl (bulk microstructure, compact mechanical hardness, ionic and electrical conductivity) are not very significant. However, since the LPSCl crystallites in contact with Li metal decompose during electrochemical cycling, the doped structure is a path toward an optimum solid electrolyte interphase (SEI). The electrochemical benefits of doping are largely an interphase effect.

X‐ray photoelectron spectroscopy (XPS) analysis was conducted to investigate the surface chemical compositions of 5LBH‐LPSCl, baseline‐LPSCl and LBH samples, with the results presented in Figure 1b,c, and Figure S2, Supporting Information. All samples were prepared inside an inert Ar atmosphere glovebox and subsequently transferred to the XPS chamber using an airtight capsule to minimize air exposure. The high‐resolution B 1s spectra in Figure 1b reveal that the LBH precursor exhibits two peaks at 187.6 and 191.9 eV, corresponding to the ‐BH_4_ and ‐BO_x_ species, respectively. The existence of ‐BO_x_ is likely due to the hydrolysis of LiBH_4_ with trace amounts of water during transfer or intrinsic impurities in the commercially purchased precursor.^[^ ^73^ ^]^ This is also evident in the ^11^B NMR spectra (Figure 1e) where a chemical shift of 1 ppm is observed. In contrast, the baseline‐LPSCl sample displays a strong XPS peak at 189.5 eV, attributed to the overlapping of P 2s (‐PS_4_) spectra. For the 5LBH‐LPSCl specimen, three peaks are observed at 187.5, 189.5, and 191.9 eV, which are assigned to ‐BH_4_, ‐PS_4_, and ‐BO_x_, respectively. Figure 1c shows the Li 1s spectra, with all samples exhibiting single peaks at 55.5, 55.3, and 55.8 eV for 5LBH‐LPSCl, baseline‐LPSCl, and LBH, respectively. Compared to baseline‐LPSCl, a positive shift in the Li 1s binding energy is observed for 5LBH‐LPSCl, which is attributed to the higher binding energy of Li 1s in LBH compared to that in baseline‐LPSCl. Figure S2, Supporting Information provides high‐resolution XPS spectra of P 2p, S 2p, and Cl 2p, showing no discernible differences in these regions, indicating minimal chemical variations for these species. Higher binding energy of Li 1s in LBH compared to that of LPSCl implies that Li atom donates more charge to BH_4_ than to halogens. This is consistent with the fact that the electron affinity of BH_4_ is higher than that of any halogen.

To further probe the structural changes induced by LBH doping, Raman spectroscopy and magic‐angle spinning (MAS) nuclear magnetic resonance (NMR) were performed. Figure 1d and Figure S3, Supporting Information present the Raman spectra of LBH‐doped LPSCl and baseline‐LPSCl. All specimens show a major peak at 423 cm^−1^ along with two smaller broad peaks at 263 and 572 cm^−1^, which originate from the P‐S tetrahedra stretching modes in LPSCl. Additionally, compared to baseline‐LPSCl, the LBH‐doped SSEs exhibit two additional peaks in the range of 2400–2600 cm^−1^, attributed to the B‐H stretching modes.^[^ ^74^ ^]^ Figure 1e,f, and Figure S4, Supporting Information display the solid‐state ^7^Li, ^11^B, and ^31^P NMR spectra of the 5LBH‐LPSCl SSE, alongside the baseline‐LPSCl and as‐received LBH powder samples to assess the incorporation of BH_4_ and identify any potential structural changes in LPSCl. Negligible differences are observed in the ^7^Li and ^31^P spectra of the doped and baseline electrolytes, indicating that the associated structural differences are minimal. A minor ^7^Li resonance at a higher frequency is observed, which may originate from Li sites in proximity to BH_4_. The ^11^B NMR spectra of the doped electrolyte and pure LiBH_4_ are nearly identical, indicating that the local BH_4_ environment is preserved.

To assess whether LBH is incorporated into the LPSCl framework, or whether it exists as a segregated phase, a ^11^B{^31^P} rotational‐echo double‐resonance (REDOR) experiment was performed on the 5LBH‐LPSCl sample.^[^ ^75^ ^]^ This experiment leads to a loss in signal amplitude in ^11^B NMR that is dependent to the dipolar recoupling time (*t*_rec_) and the direct dipolar coupling between ^11^B and ^31^P, which is solely determined by the internuclear distance. A REDOR effect would thus only be observed in the extreme intermixing case. The result from this experiment is shown in Figure S5a, Supporting Information where a strong dipolar dephasing is observed. A REDOR transform was performed using Tikhonov regularization to extract a distance distribution that maps the closest ^11^B‐^31^P distance for each BH_4_ anion (Figure S5b, Supporting Information).^[^ ^76^ ^]^ About 85% of the BH_4_ anions are at a distance of roughly 4.5 Å from a phosphorus center, indicating that there are no large LBH segregated phases and that the dopant is homogeneously mixed throughout the LPSCl material. In addition, Figure S6, Supporting Information presents cryogenic transmission electron microscopy (cryo‐TEM) coupled with electron energy loss spectroscopy (EELS) mapping of the 5LBH‐LPSCl specimen, revealing a homogenous distribution of the B signal alongside other elements such as S, Cl, and P originating from the LPSCl SSE. As will be discussed later in the manuscript, the inhomogeneous mixing of LiBH_4_ and LPSCl SSE by manual grinding results in inferior electrochemical performance.

It has been demonstrated that the undoped LPSCl microstructure (particle size/distribution, in‐turn affecting grain size, porosity and interface roughness) critically influences the electrochemical stability of the resultant SSE.^[^ ^72^, ^77^ ^]^ Figure S7a,b, Supporting Information display scanning electron microscopy (SEM) images of the 5LBH‐LPSCl and baseline‐LPSCl samples. The corresponding LPSCl particle size distribution profiles are provided in Figure S7c,d, Supporting Information, along with statistical values for the average size and standard deviation (σ). Per the SEM images the average particle sizes are 1.49 ± 0.94 µm for 5LBH‐LPSCl and 1.54 ± 0.82 µm for baseline‐LPSCl. This indicates that a comparable particle size distribution is maintained after 24 h of mechanical milling with or without the borohydride additive. Vickers hardness indentation testing was performed to assess the hardness of LPSCl compacts with and without LBH doping. As shown in Figure S8, Supporting Information, all samples exhibit similar hardness levels, measured as 912.7 ± 25.4 MPa for baseline‐LPSCl, 873.5 ± 22.3 MPa for 1LBH‐LPSCl, 995.2 ± 74.8 MPa for 5LBH‐LPSCl, and 977.5 ± 46.8 MPa for 10LBH‐LPSCl. Since the milled LPSCl microstructure and the mechanical response of the compacts are minimally influenced by LBH addition, the measured CCD changes should originate from the LiBH_4_ doping per se.

Figure 1g–i present the of ionic and electrical conductivity measurements of baseline and LBH‐doped SSEs. Ionic conductivity was determined by resistance values obtained from electrochemical impedance spectroscopy (EIS), using titanium alloy plungers as blocking electrodes. Electrical conductivity was measured in Ni/SSE/Ni symmetric cells under a constant applied voltage of 0.5 V for 2 h. As shown in Figure 1i, the measured ionic conductivities are 1.59 ± 0.13 mS cm^−1^ for baseline‐LPSCl, 1.45 ± 0.19 mS cm^−1^ for 1LBH‐LPSCl, 2.53 ± 0.4 mS cm^−1^ for 5LBH‐LPSCl, and 1.28 ± 0.25 mS cm^−1^ for 10LBH‐LPSCl. Complete substitution of Cl^−^ anions by BH_4_^−^ polyanions in argyrodite SSEs has been reported to enhance ionic conductivity due to the weaker Li‐BH_4_ interaction compared to Li‐Cl, facilitating faster Li‐ion diffusion.^[^ ^68^ ^]^ A similar trend is observed in partially BH_4_‐substituted LPSCl.^[^ ^71^ ^]^ However, excessive BH_4_ doping may increase anion disorder and lead to the precipitation of LiCl, which is a poor ionic conductor. Figure S9, Supporting Information presents the temperature‐dependent EIS Nyquist ploys and corresponding Arrhenius plots for 5LBH‐LPSCl and baseline‐LPSCl. The calculated activation energies are 0.31 eV for 5LBH‐LPSCl and 0.41 eV for baseline‐LPSCl, indicating a reduced energy barrier for ionic conduction in 5LBH‐LPSCl. This result aligns with the observed ionic conductivity trend. Electrical conductivity demonstrates negligible variation, with a slight increase as BH_4_ doping increases. While 5LBH‐LPSCl exhibits the highest ionic conductivity, the enhancement is not dramatic. Taken by itself, a ≈50% increase in ionic conductivity should not translate into a major difference in the electrochemical stability of the SSE. As will be demonstrated, factors related to the LBH modified SEI are critically important in that regard.

The role of LBH doping in LPSCl SSE electrochemical behavior was first evaluated using Li/SSE/Li symmetric cells. Polyether ether ketone (PEEK) cells were employed under a stack pressure of 9 MPa, with all measurements being conducted at room temperature unless otherwise specified. Critical current density (CCD) is a key parameter for SSEs, representing the maximum applied current before the cell undergoes electrical short‐circuiting. To determine the CCD two types of electrochemical measurements were conducted using symmetric cells. The first approach applies a constant electrodeposition/dissolution capacity while incrementally increasing the current density, thereby transferring the same amount of lithium during each cycle while progressively reducing the duration. This method helps to mitigate void accumulation at the Li/SSE interface and prevents premature short‐circuiting, which occurs when both current density and capacity are increased simultaneously.^[^ ^78^, ^79^ ^]^

**Figure 2:**
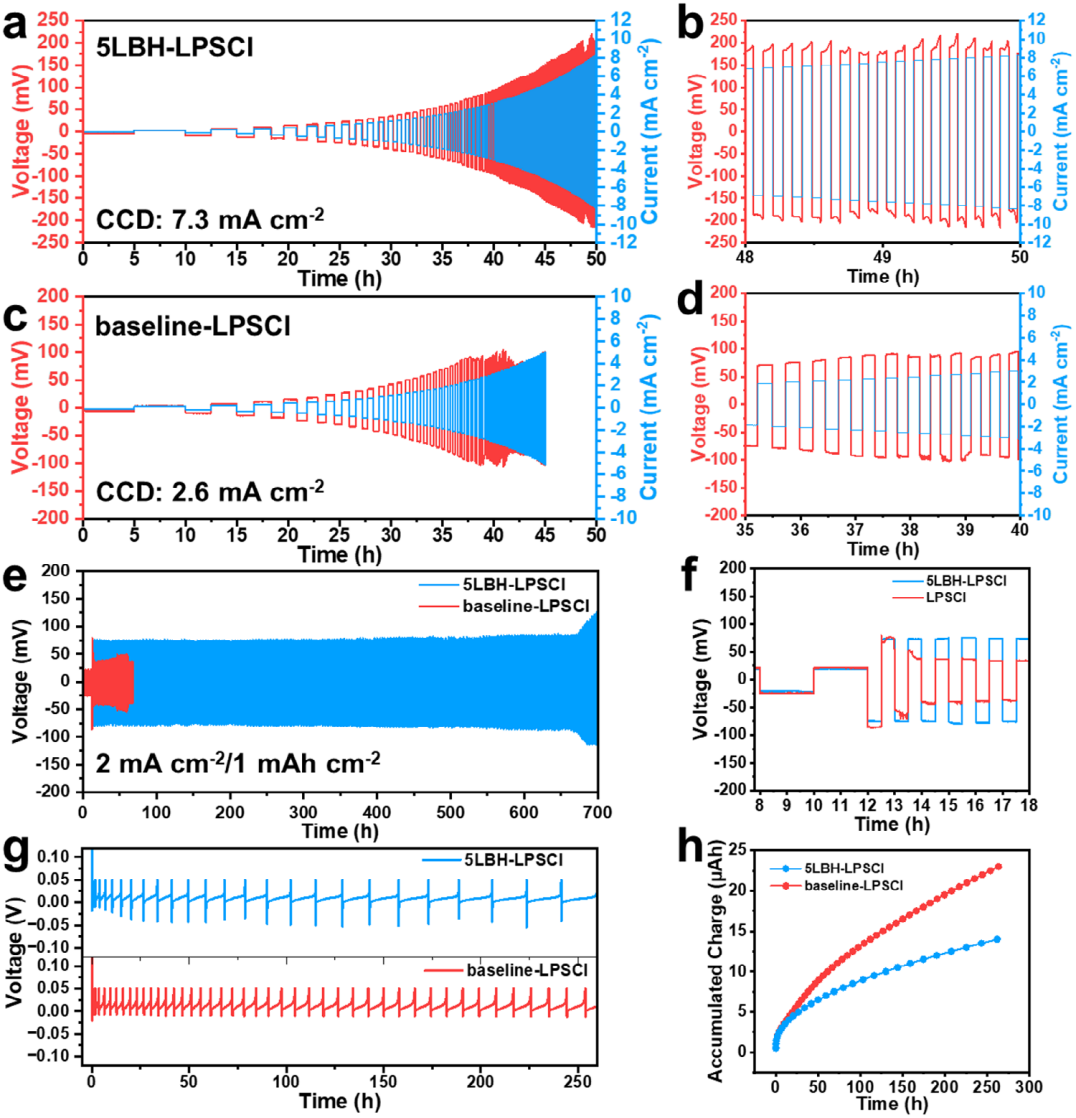
a–f) Electrochemical performance of symmetric cells based on 5LBH‐LPSCl and baseline‐LPSCl SSEs. a,c) CCD tests. b,d) Representative galvanostatic profiles when cell shorting occurs. e) Galvanostatic cycling performance, activated at 0.5 mA cm^−2^/1 mAh cm^−2^ for 3 cycles, followed by 2 mA cm^−2^/1 mAh cm^−2^ for the remainder. f) Representative galvanostatic profiles from third to ninth cycles. g,h) CTTA analysis of Li|SSE|stainless steel cells employing 5LBH‐LPSCl and baseline‐LPSCl SSE g) Voltage‐time profiles. h) Accumulated charge‐time profiles.a–f) Electrochemical performance of symmetric cells based on 5LBH‐LPSCl and baseline‐LPSCl SSEs. a,c) CCD tests. b,d) Representative galvanostatic profiles when cell shorting occurs. e) Galvanostatic cycling performance, activated at 0.5 mA cm^−2^/1 mAh cm^−2^ for 3 cycles, followed by 2 mA cm^−2^/1 mAh cm^−2^ for the remainder. f) Representative galvanostatic profiles from third to ninth cycles. g,h) CTTA analysis of Li|SSE|stainless steel cells employing 5LBH‐LPSCl and baseline‐LPSCl SSE g) Voltage‐time profiles. h) Accumulated charge‐time profiles.

Per this protocol a constant electrodeposition/dissolution capacity of 0.5 mAh cm^−2^ was applied with an initial current density of 0.1 mA cm^−2^, followed by stepwise increments of 0.1 mA cm^−2^ until short‐circuiting occurred. At each current density, the electrodeposition/dissolution duration was adjusted to reach the target capacity of 0.5 mAh cm^−2^ (e.g., 5 h at 0.1 mA cm^−2^ and 2.5 h at 0.2 mA cm^−2^). In sulfide‐based SSEs, both “hard” and “soft” short circuiting can occur.^[^ ^80^, ^81^ ^]^ Hard breakdown is easily detected by a sudden drop in overpotential, whereas soft breakdown, resulting from mixed ion/electron conduction, is often subtle. Any reduction in overpotential compared to the previous cycle is considered indicative of a soft breakdown and, therefore, short‐circuiting. **Figures** **2**a–d and S10, Supporting Information provide CCD measurements for LPSCl samples with and without LBH doping. The baseline‐LPSCl SSE exhibits a CCD of 2.6 mA cm^−2^, consistent with previous reports for a microstructurally optimized but undoped electrolyte.^[^ ^72^ ^]^ By contrast, the 5LBH‐LPSCl SSE achieves a CCD of 7.3 mA cm^−2^, nearly three times higher. The CCD values are 2.7 and 5.1 mA cm^−2^ for 1LBH‐LPSCl and 10LBH‐LPSCl, respectively, indicating performance improvements over the baseline but still inferior to 5LBH‐LPSCl.

In the second type of CCD measurement, a constant time was applied during each electrodeposition/dissolution, resulting in the simultaneous increase of current density and capacity. In this protocol, the cells were cycled starting at 0.1 mA cm^−2^, with a step size of 0.1 mA cm^−2^ and a fixed duration of 1 h per step. Figure S11, Supporting Information displays the galvanostatic profiles of 5LBH‐LPSCl and baseline‐LPSCl samples. The baseline‐LPSCl SSE experienced short‐circuiting at 2.1 mA cm^−2^, whereas the 5LBH‐LPSCl maintained stable cycling until 3.0 mA cm^−2^. Such electrochemical performance is among the most favorable per Table S3, Supporting Information. As will be demonstrated, the improvement results not only from incrementally increased ionic conductivity, but (more importantly) from the formation of chemically stable, electrically insulating, and ionically conductive SEI.

To further evaluate the effectiveness of LiBH_4_ doping, other lithium polyanionic salts were also tested as doping agents for LPSCl. The identically co‐milled and tested additives were LiBF_4_ and LiPF_6_, which are commonly employed in liquid electrolytes. As shown in Figure S12, Supporting Information, the ionic conductivity of 5LBF‐LPSCl and 5LPF‐LPSCl was measured to be 0.39 and 0.72 mS cm^−1^, respectively. This is lower than that of 5LBH‐LPSCl (2.53 mS cm^−1^) and even lower than baseline LPSCl (1.59 mS cm^−1^). The reduction in ionic conductivity is likely associated with reactivity between LiBF_4_/LiPF_6_ and LPSCl, as predicted by the Materials Project, with the corresponding reaction pathways and reaction energies summarized in Table S1, Supporting Information.^[^ ^60^ ^]^ Figure S13, Supporting Information presents the high‐resolution F 1s spectra of the 5LBF‐LPSCl and 5LPF‐LPSCl SSEs. Both samples exhibit two peaks with the dominant peak centered at 684.7 eV, corresponding to LiF. The secondary peak at 686.3 eV in the 5LBF‐LPSCl sample is attributed to LiBF_4_ while the peak at 687.2 eV in the 5LPF‐LPSCl sample is ascribed to LiPF_6_.^[^ ^82^ ^]^ The pronounced LiF signals in both samples indicate substantial electrolyte decomposition during the milling process. Since LiF possesses inherently low ionic conductivity, its formation in the bulk of the electrolyte will be deleterious.

Figure S14, Supporting Information plots the galvanostatic CCD test profiles for 5LBF‐LPSCl and 5LPF‐LPSCl, with the CCD values being 1.3 and 1.6 mA cm^−2^, respectively. Additionally, LiBO_2_‐doped LPSCl is synthesized using a similar approach and denoted as 5LBO‐LPSCl. As shown in Figure S15, Supporting Information, 5LBO‐LPSCl exhibits a CCD of 2.1 mA cm^−2^, even lower than baseline‐LPSCl (2.6 mA cm^−2^). These results demonstrate that LiBF_4_, LiPF_6_, LiBO_2_ doping negatively impact the CCD of LPSCl SSEs, underscoring the necessity of selecting a doping agent that is thermodynamically compatible with LPSCl. Consequently, 5LBH‐LPSCl and baseline‐LPSCl were selected as the systems for in‐depth analytical examination.

Figure 2e presents the cycling performance of the symmetric cells, comparing 5LBH‐LPSCl with baseline SSEs. The cells were initially activated at 0.5 mA cm^−2^ with a capacity of 1 mAh cm^−2^ for three cycles, followed by continuous cycling at 2 mA cm^−2^ with the same capacity for the reminder of the test. Figure 2f provides enlarged profiles from the third to the ninth cycle. The baseline‐LPSCl exhibited cell failure just one cycle after the activation process, short‐circuiting after 13 h (4 cycles). By contrast, the 5LBH‐LPSCl sample displayed stable cycling up to 700 h (692 cycles) without short‐circuiting. These differences in cycling stability result in variations in cumulative capacity. The baseline‐LPSCl displayed a cumulative capacity of 4 mAh cm^−2^, corresponding to a lithium electrodeposition/dissolution thickness of 20 microns. In contrast, the 5LBH‐LPSCl specimen demonstrated a cumulative capacity of 692 mAh cm^−2^, with an associated lithium electrodeposition/dissolution thickness of 3460 microns.

Figure S16, Supporting Information plots the EIS Nyquist curves for Li|5LBH‐LPSCl|Li and Li|baseline‐LPSCl|Li symmetric cells after various cycles. The plots were fitted using ZSimpWin software with the corresponding results summarized in Table S2, Supporting Information. The 5LBH‐LPSCl sample exhibited a lower combined SEI and charge‐transfer resistance (R_SEI_+R_CT_) than baseline‐LPSCl before and after the three‐cycle activation process. Specifically, the R_SEI_+R_CT_ values were 2.9 and 5.3 Ω for 5LBH‐LPSCl, compared to 7.4 and 7.9 Ω for baseline‐LPSCl. At an increased current density of 2 mA cm^−2^, a sudden drop in bulk (R_b_) and interfacial resistance (R_SEI_+R_CT_) was observed for baseline‐LPSCl starting from the fourth cycle, indicating short circuiting. This abrupt (rather than gradual) decrease in R_b_ suggests that electronic conduction through metal dendrite became dominant, while ionic conduction through the SSE became secondary. This phenomenon is further evidenced by the sharp decrease in overpotential in Figure 2f.

By contrast, the 5LBH‐LPSCl specimen exhibited stable interfacial resistance with only a slight decrease of R_b_, likely due to improved interfacial contact between cell components. Figure S17, Supporting Information shows cycling performance at a lower current of 1 mA cm^−2^ and a capacity of 1 mAh cm^−2^. Both cells demonstrated stable cycling until 702 h (351 cycles), after which the baseline‐LPSCl SSE exhibited a sudden voltage drop, indicating a short circuit. By contrast, the 5LBH‐LPSCl SSE continued stable cycling for up to 1200 h (600 cycles) with no signs of cell shorting. The cumulative capacity reached 351 mAh cm^−2^ for baseline‐LPSCl and 600 mAh cm^−2^ for 5LBH‐LPSCl, corresponding to cumulative lithium electrodeposition/dissolution thickness of 1755 and 3000 µm, respectively. Furthermore, the 5LBH‐LPSCl sample exhibited a lower overpotential during cycling compared to baseline‐LPSCl, indicating enhanced electrodeposition/dissolution kinetics.

The electrochemical performance of 5LBH‐LPSCl and baseline‐LPSCl was further evaluated using Li|SSE|Cu asymmetric half‐cells. Figure S18, Supporting Information shows the galvanostatic cycling profiles along with Coulombic efficiency (CE), tested at 1 mA cm^−2^ with an electrodeposition capacity of 1 mAh cm^−2^ and an electrodissolution cutoff voltage of 0.2 V. The initial Coulombic efficiency (ICE) values were comparable between the two samples, measuring 96.1% for 5LBH‐LPSCl and 96.7% for baseline‐LPSCl. However, the Li|baseline‐LPSCl|Cu cell experienced significant CE fluctuations after 15 cycles, whereas the Li|5LBH‐LPSCl|Cu cell maintained stable cycling for up to 50 cycles, with an average cycling CE of 96.7%.

To further evaluate the stability of Li metal against different SSEs, Coulometric titration time analysis (CTTA) was performed employing Li|SSE|stainless steel half‐cells. The experimental protocol followed a previous report.^[^ ^83^ ^]^ Briefly, during each Coulometric titration period, a small charge was applied at 0.01 mA cm^−2^ for 10 min. Theoretically, this would result in lithium deposition with a capacity of 1.67 µAh cm^−2^ (≈8 nm thickness). If there were no parasitic reactions at either electrode, the open circuit voltage (OCV) would approach zero. In reality, a SEI layer will be already present at the (counter electrode) Li foil‐SSE interface, and will spontaneously form at the working electrode once electrodeposition occurs. The formation of Li_2_S, Li_3_P, Li_3_PS_4_, LiCl, etc. is thermodynamically favored, leading to an increase in OCV as the reactions proceed on the working electrode. Since these SEI phases are electrical insulators (Li_3_P has some conductivity), when the electrodeposit is fully reacted, the voltage will spike to the 50 mV cutoff. Once this occurs, the next titration step is applied and the process is repeated. The voltage and accumulated charge versus time profiles are shown in Figure 2g,h. Over 260 h of testing, the cell with 5LBH‐LPSCl SSE accumulates less charge (14 µAh) than the baseline‐LPSCl (23 µAh), indicating a more stable interface with fewer parasitic reactions between the Li metal and the SSE.

The electrochemical performance at low temperatures was evaluated using Li|SSE|Li symmetric cells. As shown in Figure S19a–d, Supporting Information, at 6 °C, the 5LBH‐LPSCl SSE exhibits a CCD of 2.0 mA cm^−2^, versus the baseline‐LPSCl which sustains 0.9 mA cm^−2^. Both values are lower than those at room temperature, reflecting the sluggish Li‐ion kinetics at reduced temperatures. Figure S19e,f, Supporting Information show the cycling performance of symmetric cells employing 5LBH‐LPSCl and baseline‐LPSCl SSEs, tested at 0.5 mA cm^−2^ with a capacity of 0.25 mAh cm^−2^. The 5LBH‐LPSCl cell maintains stable cycling for over 500 h (500 cycles), while the baseline‐LPSCl cell short‐circuits after the first cycle, highlighting the superior interfacial stability of 5LBH‐LPSCl at low temperatures. Further tests at an even lower temperature of −14 °C (Figure S20, Supporting Information) at 0.2 mA cm^−2^ and 0.2 mAh cm^−2^ reveal that the baseline‐LPSCl cell fails at cycle 2, whereas the 5LBH‐LPSCl cell continues stable operation for over 500 h (250 cycles). These results demonstrate the markedly enhanced low‐temperature performance of 5LBH‐LPSCl SSE, indicating its potential for applications under extreme cold conditions.

To understand the role of mechanical milling versus conventional mechanical mixing in resultant structure/performance, a series of experiments were conducted where the LBH precursor was combined with LPSCl using mortar and pestle. The as‐derived material was labelled 5LBH/LPSCl. Figure S21a–c, Supporting Information provide the XRD and XPS characterizations of the 5LBH/LPSCl sample. No major differences were observed in the XRD patterns versus 5LBH‐LPSCl. However, XPS analysis indicates a lower intensity of B signals compared to that of 5LBH‐LPSCl. This is likely caused by the inhomogeneous distribution of LBH in the composite material. Figure S21d,e, Supporting Information present the electrochemical measurement for the 5LBH/LPSCl sample. The symmetric cell has a CCD of 2.2 mA cm^−2^, much lower than that of 5LBH‐LPSCl (7.3 mA cm^−2^). This indicates that employing mechanical milling is critical for achieving effective LBH doping.

**Figure 3:**
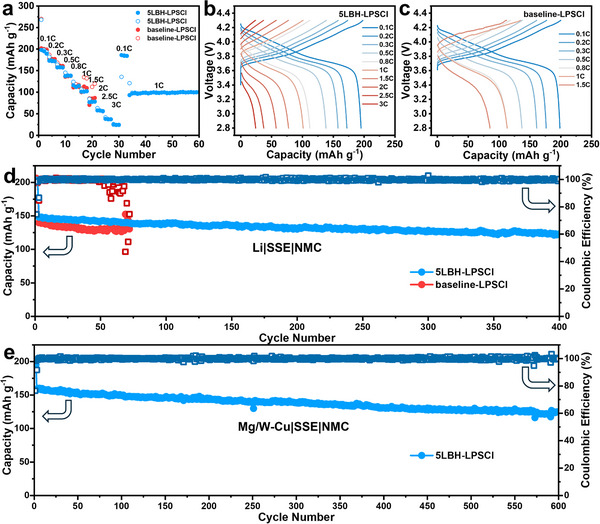
a) Rate capability of NMC full cells using 5LBH‐LPSCl and baseline‐LPSCl SSEs. b,c) Representative galvanostatic profiles at different current densities. d) Cycling performance of Li|SSE|NMC full cells, tested at 0.5C after initial 2 cycles at 0.1C. e) Cycling performance of Mg/W‐Cu|SSE|NMC AF‐ASSB employing 5LBH‐LPSCl SSE, tested at 0.33C after initial 2 cycles at 0.1C.

The electrochemical performance of baseline‐LPSCl and 5LBH‐LPSCl SSEs was further analyzed in full‐cell configurations. The composite cathode was prepared by mixing commercially purchased lithium niobate (LiNbO_3_) coated LiNi_0.8_Co_0.1_O_2_ (NMC811), LPSCl SSE, and carbon fiber (VGCF) in a weight ratio of 7:3:0.3. A voltage window of 2.8 – 4.3 V was employed for the test. **Figure** **3**a presents the rate performance of ASSBs with either 5LBH‐LPSCl or baseline‐LPSCl SSEs. The cells underwent five cycles at each rate from 0.1C to 3C (1C = 200 mA g^−1^), followed by a return to 0.1C, and then continuous cycling at 1C for 25 cycles. Figure 3b,c display the galvanostatic charge/discharge data at each rate. The 5LBH‐LPSCl cell demonstrates superior rate capability, delivering 194, 172, 158, 138, 114, 102, 77, 57, 38, and 24 mAh g^−1^ at 0.1C, 0.2C, 0.3C, 0.5C, 0.8C, 1C, 1.5C, 2C, 2.5C, and 3C, respectively. By contrast, the baseline‐LPSCl cell short‐circuits at 1C during cycle 17. Figure 3d presents the cycling stability of ASSBs employing both SSEs. Cells were conditioned at 0.1C for two cycles before testing at 0.5C. The 5LBH‐LPSCl cell exhibits stable cycling up to 400 cycles, retaining 83% of its initial capacity (from 148 mAh g^−1^ at cycle 3 to 123 mAh g^−1^ at cycle 400) with a low decay rate of 0.04% per cycle. By contrast, the baseline‐LPSCl cell undergoes stable cycling for only 55 cycles before short‐circuiting, as evidenced by fluctuating CEs from cycle 56 onward.

As a proof of concept, anode‐free all solid‐state batteries (AF‐ASSBs) were assembled and tested with 5LBH‐LPSCl SSE. Conventional copper current collector coated by a bilayer of 140 nm magnesium / 20 nm tungsten (termed Mg/W‐Cu) was employed as the support. It is known that Mg facilitates wetting of Li metal, while the W prevents the corrosion of the Cu by the SSE.^[^ ^35^, ^36^, ^84^, ^85^ ^]^ Figure 3e shows the cycling performance of the Mg/W‐Cu|5LBH‐LPSCl|NMC AF‐ASSB. Figure S22, Supporting Information provides the associated galvanostatic profiles at select cycle numbers. The cell underwent an activation process at 0.1C for two cycles before switching to 0.33C. In a standard test, a mass loading of ≈1.5 mAh cm^−2^ was used, with 1C corresponding to an areal current density of 1.5 mA cm^−2^. Electrochemical measurements were conducted within a voltage range of 2.8–4.3 V at room temperature. The Mg/W‐Cu|5LBH‐LPSCl|NMC cell delivers charge/discharge capacities of 262/200 mAh g^−1^, corresponding to an initial Coulombic efficiency (ICE) of 76%. Upon switching to 0.33C, the cell initially exhibits a reversible capacity of 159 mAh g^−1^, followed by stable cycling over 600 cycles, maintaining a capacity of 125 mAh g^−1^. The resultant capacity retention is 79%, with a decay rate of 0.04% per cycle. The above electrochemical measurements confirm the enhanced performance of 5LBH‐LPSCl SSE. In order to test the practicality of the 5LBH‐LPSCl SSE, a high mass‐loading NMC cathode was employed with an areal capacity exceeding 6 mAh cm^−2^, higher than commercial lithium‐ion batteries cathode (4 mAh cm^−2^). As shown in Figure S23, Supporting Information, the Mg/W‐Cu|5LBH‐LPSCl|NMC cell delivers initial charge/discharge capacities of 6.5/4.9 mAh cm^−2^, corresponding to an ICE of 75%. After switching to a higher current of 0.33C, the cell stably cycled for more than 100 cycles, exhibiting a capacity retention of 92.4%. As summarized in Table S4, Supporting Information, these electrochemical properties are quite promising relative to previous studies with state‐of‐the art performance.

Cryogenic focused ion beam (cryo‐FIB) SEM was employed to investigate the post‐cycling metal/SSE interface morphology in Li symmetric cells using either 5LBH‐LPSCl or baseline LPSCl SSEs. The cells were cycled for 100 times at 1 mA cm^−2^ and 1 mAh cm^−2^, after which the cells were disassembled in an inert glovebox. Figure S24a,d, Supporting Information present images of the fractured surfaces of Li|5LBH‐LPSCl|Li and Li|baseline‐LPSCl|Li cells, respectively. Figure S24b,e, Supporting Information highlight cryo‐FIB cross‐sectional SEM images of the Li/SSE interface in the electrodeposited state. The corresponding energy dispersive X‐ray spectroscopy (EDXS) maps provided in Figure S24c,f, Supporting Information. Analysis of the fractured surface reveals that the baseline‐LPSCl pellet developed cracks and was separated by a layer of Li metal. It may be hypothesized that the cracks in the baseline‐LPSCl are caused by the stresses from nonuniform Li electrodeposition/dissolution through the thick SEI layer. Once SSE fracture occurs, Li metal can wet the crack interiors, and if remain electrochemically active, would further drive crack opening at every cycle.^[^ ^72^ ^]^ The crack direction is observed to be ≈75° from the applied uniaxial pressure, deviating from the expected maximum shear stress orientation of 45° relative to the applied uniaxial stress direction. Conversely, the post‐cycled 5LBH‐LPSCl sample displayed no visible signs of metal penetration or of cracking. The thinner and more ionically conductive SEI layer would homogenize the ion flux during electrodeposition/dissolution, reducing localized stresses at the metal‐SSE interface. In the 5LBH‐LPSCl sample the metal‐SSE interface remained uniform and in intimate contact, which would minimize current constriction during cycling.^[^ ^86^ ^]^ In the baseline‐LPSCl cell, the Li deposits were interspersed with isolated SSE particles, as evidenced by the presence of P, S, and Cl signals in the EDXS maps. This could also be attributed to localized stresses that promote SSE fracture, allowing the Li metal to wet the fracture surfaces thereby “flowing” around individual particles. Kirkendall type voiding in the Li metal are also a symptom of non‐uniform electrodeposition/dissolution, as well as of inhomogeneous reactivity of the Li and the SSE.^[^ ^41^ ^]^

**Figure 4:**
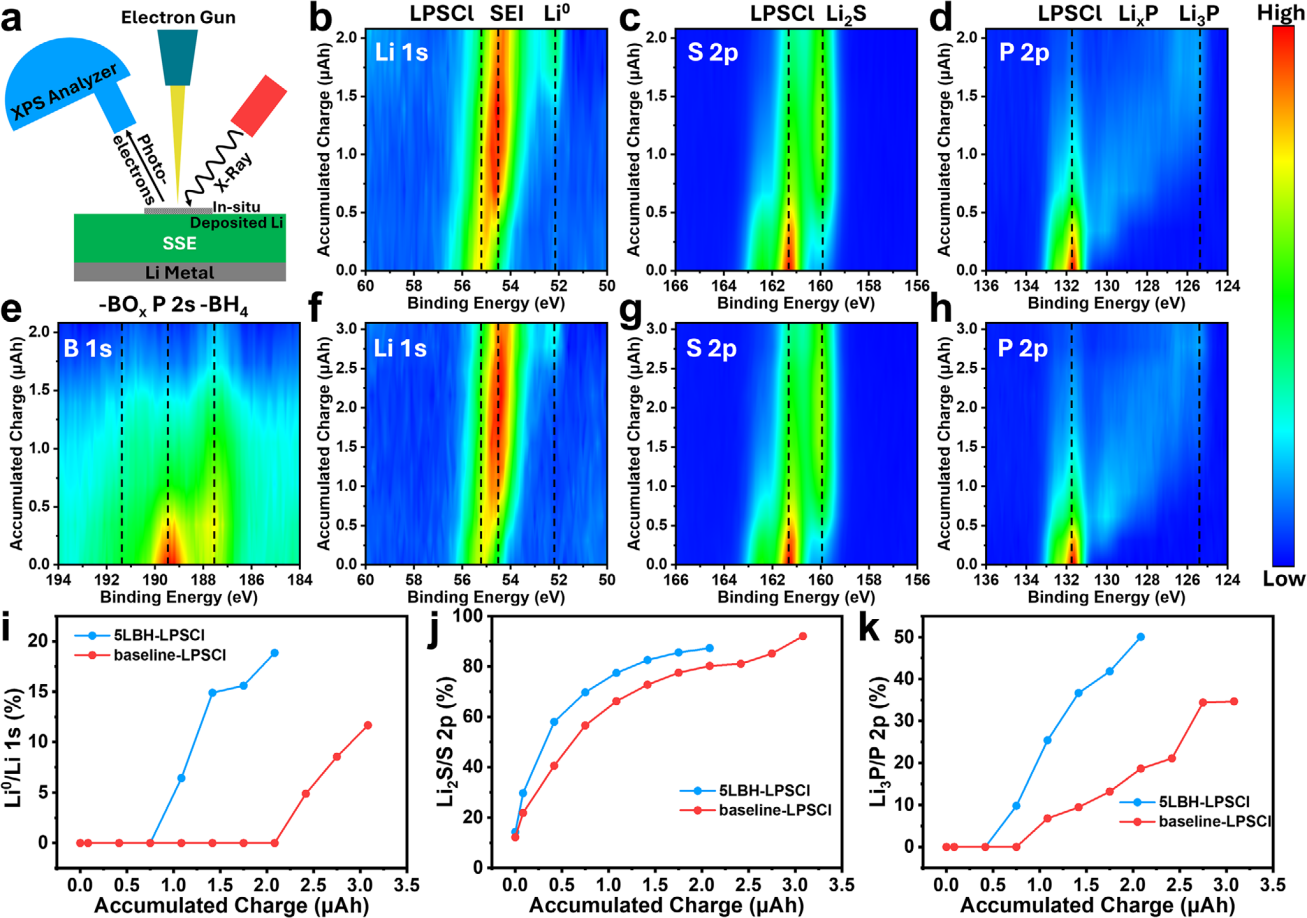
In situ XPS measurements of Li electrodeposition at the SSE surface. a) Schematic illustration of the experimental setup. b–e) Contour plots of Li 1s, S 2p, P 2p, and B 1s spectra for 5LBH‐LPSCl. f,g) Contour plots of Li 1s, S 2p, and P 2p spectra for baseline‐LPSCl. Quantification of XPS spectra of i) metallic Li (Li^0^) in Li 1s, j) Li_2_S in S 2p, and k) Li_3_P in P 2p.In situ XPS measurements of Li electrodeposition at the SSE surface. a) Schematic illustration of the experimental setup. b–e) Contour plots of Li 1s, S 2p, P 2p, and B 1s spectra for 5LBH‐LPSCl. f,g) Contour plots of Li 1s, S 2p, and P 2p spectra for baseline‐LPSCl. Quantification of XPS spectra of i) metallic Li (Li^0^) in Li 1s, j) Li_2_S in S 2p, and k) Li_3_P in P 2p.

To further investigate the role of metal/SSE interface and differences in SEI compositions between the baseline and 5LBH‐LPSCl specimens, in situ XPS Li electrodeposition experiments were conducted, with results presented in **Figure** **4**. Figure 4a illustrates the experimental setup, designed according to previous studies on the surface composition evolution and Li metal deposition in sulfide‐based SSEs using in situ XPS techniques.^[^ ^87^, ^88^, ^89^ ^]^ Detailed experimental procedures can be found in the Experimental Section. Briefly, SSE compacts with Li metal were prepared and transferred to the XPS chamber without air exposure. The sample was mounted on an XPS sample stage using conductive carbon that connects to the ground with the exposed LPSCl surface facing the X‐ray beam. The surface was initially cleaned using an Ar cluster gun (2.5 keV) to remove adventitious carbon contamination. A low‐energy electron neutralizer was employed as the electron source with the current fixed at 5 µA and the accumulated charge calculated as the product of current and exposure time. Figure 4b–e display the XPS contour plots of Li 1s, S 2p, P 2p, and B 1s spectra for 5LBH‐LPSCl at a cumulative charge of 2.08 µAh. Figure 4f–h provide the Li 1s, S 2p, and P 2p spectra for baseline‐LPSCl with a total charge of 3.08 µAh. Figure S25, Supporting Information provides the Cl 2p spectra for both specimens. Figure 4i–k shows the quantitative analysis of representative components, including metallic Li (Li^0^), Li_2_S, and Li_3_P in the Li 1s, S 2p, and P 2p regions, respectively.

Figure 4b,f illustrates the evolution of Li 1s spectra for 5LBH‐LPSCl and baseline‐LPSCl. In both cases, the peak broadens and gradually shifts to lower binding energies as the accumulated charge increases, indicative of LPSCl decomposition into products such as Li_2_S, Li_x_P, and LiCl. This reaction is driven by the thermodynamic instability of LPSCl against Li metal, resulting in reductive decomposition starting at voltages below 1.7 V versus Li/Li^+^. For 5LBH‐LPSCl, after an accumulated charge of 1.1 µAh, a new peak at low binding energy (≈52.2 eV) that corresponds to the characteristic peak of metallic Li (Li^0^) appears, and continues to intensify as the charge increases. By contrast, for baseline LPSCl the Li^0^ peak does not emerge until the accumulated charge exceeds 2.4 µAh, indicating slower Li metal nucleation and growth. Figure 4i quantifies this process, showing the relative intensity of metallic Li° compared to LPSCl and Li‐containing SEI components. The accelerated formation of a metallic Li layer on 5LBH‐LPSCl suggests the development of a faster kinetically stabilized SEI and better Li wetting on its surface compared to baseline‐LPSCl. This improved wetting behavior facilitates more uniform Li nucleation and growth, reducing interfacial resistance and enhancing electrochemical performance.

The S 2p and P 2p spectra in Figure 4c,d,g,h reveal the evolution of SEI chemistry, with quantitative results presented in Figure 4j,k. In the S 2p region, the peak corresponding to the Li_2_S (S 2p_3/2_, ≈160.0 eV) intensifies with increasing accumulated charge while the peak associated with pristine LPSCl (S 2p_3/2_, ≈161.3 eV) diminishes. Quantitative analysis indicates that both samples experience rapid Li_2_S formation in the initial stage of experiment and gradually stabilize thereafter. However, 5LBH‐LPSCl exhibits a slightly faster Li_2_S growth rate than baseline‐LPSCl. In the P 2p region, both samples display a strong P 2p_3/2_ peak at ≈131.7 eV, corresponding to the P‐S tetrahedron in LPSCl. Upon reaction with electrodeposited Li metal, LPSCl undergoes stepwise reduction through intermediate Li_x_P phases before reaching the terminal Li_3_P phase (P 2p_3/2_, ≈125.4 eV), resulting in a broad binding energy distribution. In general, both P 2p spectra exhibit a gradual shift to lower binding energies and an intensity reduction as charge accumulates. As will be discussed, a P‐rich SEI layer forms near the SSE while a S‐rich SEI layer develops adjacent to the electrodeposited Li metal. Given the surface sensitivity of XPS (≈5 nm analysis depth), the overall intensity of P 2p spectra is lower than that of Li 1s and S 2p.

Figure 4k shows that Li_3_P formation occurs more rapidly on 5LBH‐LPSCl, aligning with its accelerated metallic Li^0^ deposition. Figure 4e displays the contour plot of B 1s spectra for the 5LBH‐LPSCl specimen, with three major components being identified: ‐BH_4_, P 2s, and ‐BO_x_. As previously noted, ‐BO_x_ primarily originates from borohydride hydrolysis and gradually shifts to lower binding energies with charge accumulation. The borohydride peak intensifies upon electron exposure, indicating the formation of a BH_4_‐rich SEI. The P 2s peak remains largely unchanged due to its low sensitivity to chemical state variations compared to P 2p. All B 1s signals progressively weaken with increasing charge, likely due to metallic Li and associated SEI coverage. Figure S25, Supporting Information presents Cl 2p spectra analysis for both samples. Although LPSCl decomposition leads to LiCl formation, no significant differences are observed, as the binding energies of Cl in LPSCl and LiCl are nearly identical. Their intensities of these peaks gradually decline with increasing charge accumulation.

**Figure 5:**
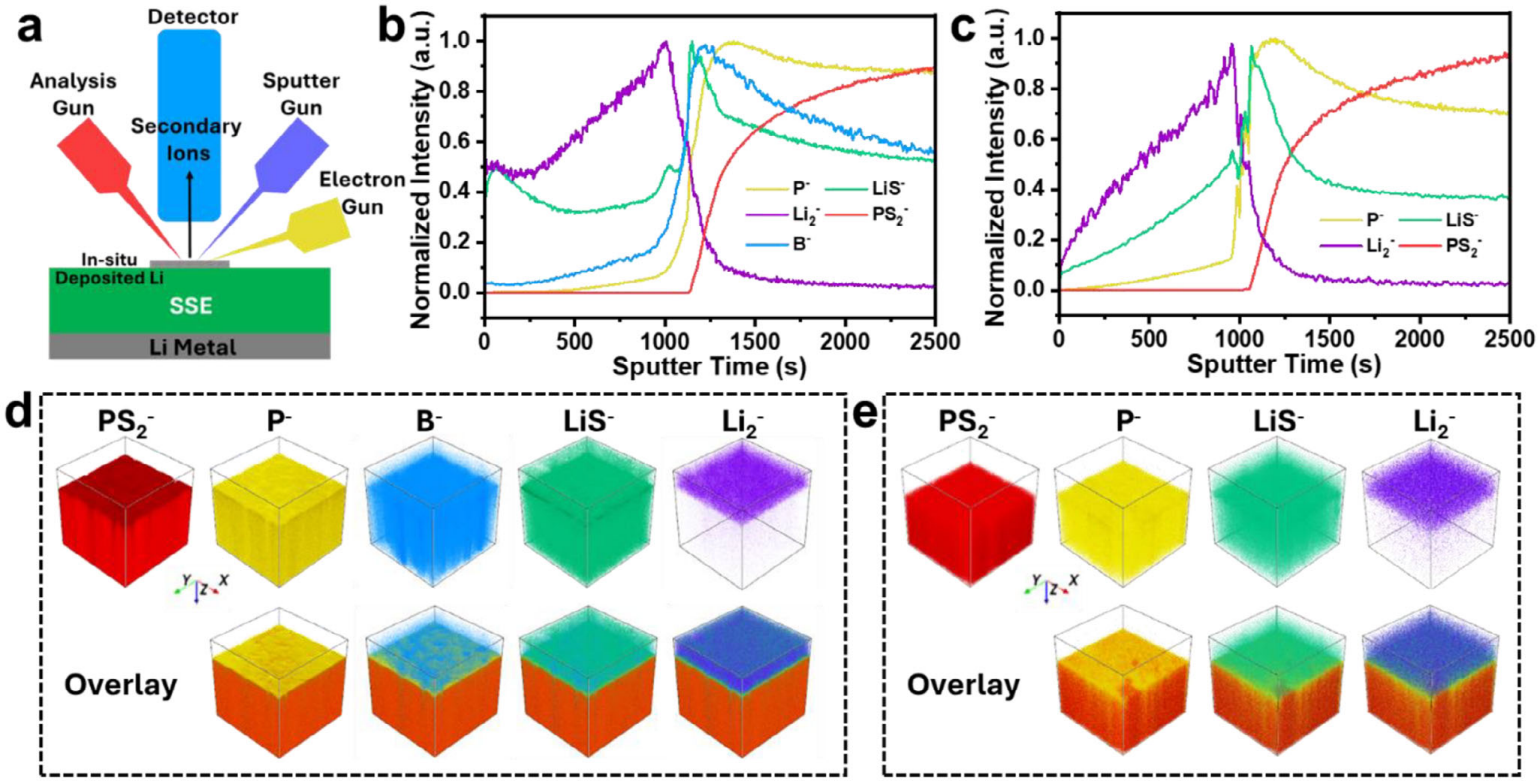
In situ TOF‐SIMS measurements of Li electrodeposition at the SSE surface. a) Schematic illustration of the experimental setup. TOFSIMS depth profiles of b) 5LBH‐LPSCl and c) baseline‐LPSCl. 3D rendering of the depth profiles shown in (b) and (c) for d) 5LBH‐LPSCl and e) baseline‐LPSCl, respectively.In situ TOF‐SIMS measurements of Li electrodeposition at the SSE surface. a) Schematic illustration of the experimental setup. TOFSIMS depth profiles of b) 5LBH‐LPSCl and c) baseline‐LPSCl. 3D rendering of the depth profiles shown in (b) and (c) for d) 5LBH‐LPSCl and e) baseline‐LPSCl, respectively.

To further investigate differences in the metal/SSE interface between 5LBH‐LPSCl and baseline LPSCl, time‐of‐flight secondary ion mass spectrometry (TOF‐SIMS) was performed with the results shown in **Figure** **5**. Figure 5a provides a schematic of the experimental setup. Analogous to the XPS measurements, in situ Li electrodeposition was conducted using a low‐energy flood electron gun (typically used to neutralize surface charges during data acquisition) at 21 eV with a beam current of ≈25 µA. Depth profiling was performed through the electrodeposited Li until reaching a certain depth into the bulk SSE. Figure S26, Supporting Information presents optical images of the 100 µm × 100 µm selected region for depth filing on 5LBH‐LPSCl before and after in situ Li electrodeposition, where the removal of metallic Li upon sputtering reveals the underlying bulk SSE.

The normalized to maximum intensity depth profiles of several species of interest for 5LBH‐LPSCl and baseline LPSCl are displayed in Figure 5b,c, respectively. The PS_2_^−^ fragment corresponds to the bulk SSE, with a rapid intensity increase indicating exposure of the substrate. The Li_2_^−^ species rather than Li^−^ was selected to differentiate electrodeposited metallic Li from Li‐containing SEI and SSE. The LiS^−^ and P^−^ fragments originate from both SEI components (e.g. Li_2_S and Li_3_P) and the SSE. At the initial stage of sputtering, species such as Li_2_^−^ and LiS^−^ are detected, indicating the removal of surface electrodeposited Li and its associated SEI. After sputtering 1130 s for 5LBH‐LPSCl and 1050 s for baseline LPSCl SSE, the PS_2_^−^ signal starts to appear and rapidly increases, marking the onset of bulk LPSCl SSE exposure. This defines the interfacial region between electrodeposited Li metal and SSE. For baseline‐LPSCl, it is observed that the maximum of the LiS^−^ signal is reached first, followed by the P^−^ signal, indicating a layered SEI structure at the interface.

Figure 5e provides the individual and overlapping 3D rendering of these species, which further confirms that a P‐rich layer forms adjacent to the SSE, followed by an S‐rich layer toward the Li metal layer consistent with prior studies.^[^ ^90^ ^]^ As shown in Figure 5b, the 5LBH‐LPSCl sample exhibits similar depth profiles for the LiS^−^ and P^−^ signals, with LiS^−^ peaking near Li_2_^−^ and P^−^ peaking adjacent to PS_2_^−^. By contrast, an additional B^−^ signal appears between the LiS^−^ and P^−^ profiles, indicating the presence of a B‐rich layer situated between the P‐rich and S‐rich layers in the SEI structure. The 3D representation in Figure 5d further visualizes the spatial distribution of the bulk SSE, SEI layers, and electrodeposited Li metal. It reveals a P‐rich layer adjacent to the SSE, an S‐rich layer near the electrodeposited Li metal, and a B‐rich layer positioned between them. This structural distinction indicates that, compared to baseline‐LPSCl, the 5LBH‐LPSCl sample develops a distinct B‐rich layer in the SEI. This layer forms as a result of the decomposition of LBH‐doped LPSCl, contributing to the observed differences in interfacial characteristics.

**Figure 6:**
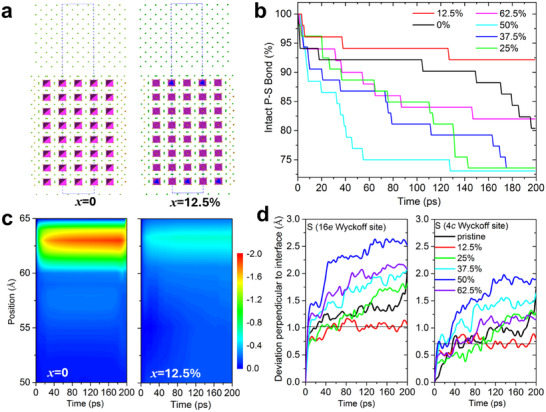
Explicit modeling for the Li‐Li_6_PS_5_(BH_4_)_x_Cl_1−x_ interfaces. a) Model structures of the pristine (*x* = 0) and BH_4_‐containing (*x* = 0.125 or 12.5%) interfaces as the key examples, with the PS_4_^3−^ units in purple tetrahedra, BH_4_^−^ in blue tetrahedra, Li in green, S in yellow, and Cl in orange. b) Percentage of the intact P‐S bonds according to the statistical analysis on the AIMD data for *x* = 0, 12.5, 25, 37.5, 50, 62.5%. c) Calculated electron transfer (color bar in *e*) from Li metal to P in the SSEs. The top position (≈65 Å) corresponds the surface of the Li metal in contact with the SSE. d) Change of atomic distribution across the interface measured by the calculated change of atomic deviation (*σ* in **Eq**. **1**), with 16e S.Explicit modeling for the Li‐Li_6_PS_5_(BH_4_)_x_Cl_1−x_ interfaces. a) Model structures of the pristine (*x* = 0) and BH_4_‐containing (*x* = 0.125 or 12.5%) interfaces as the key examples, with the PS_4_^3−^ units in purple tetrahedra, BH_4_^−^ in blue tetrahedra, Li in green, S in yellow, and Cl in orange. b) Percentage of the intact P‐S bonds according to the statistical analysis on the AIMD data for *x* = 0, 12.5, 25, 37.5, 50, 62.5%. c) Calculated electron transfer (color bar in *e*) from Li metal to P in the SSEs. The top position (≈65 Å) corresponds the surface of the Li metal in contact with the SSE. d) Change of atomic distribution across the interface measured by the calculated change of atomic deviation (*σ* in **Eq**. **1**), with 16e S.

To gain deeper insights into the impact of the doped BH_4_^−^ cluster on the stability of the Li‐SSE metal interface, interface analysis based on ab initio molecular dynamics (AIMD) was carried out on the Li‐SSE‐Li interface models (see Experimental Section for more detail), as shown in **Figure** **6**a. The interface stability can be quantified by monitoring the completeness of the PS_4_^3−^ units at the interface over the simulation time.^[^ ^66^ ^]^ While the anion cluster BH_4_^−^ is stable against Li metal,^[^ ^66^ ^]^ the interface instability of the SSE against Li metal is due to the disintegration of the PS_4_^3−^ units inside the SSE, as shown by the continuing P‐S bond breaking in Figure 6b. It is found that such disintegration in the BH_4_‐based SSE may be more or less favorable versus for unmodified LPSCl. While *x* = 0.125 mol% BH_4_^−^ substitution in (BH_4_)_x_Cl_1−x_ (i.e. 1 out of 8 Cl^−^ sites) improved the thermodynamic stability of the PS_4_^3−^ units, higher levels at 25%, 37.5%, 50%, 62.5% actually further destabilized the structure versus Li metal. In all cases, however, the PS_4_^3−^ units remained thermodynamically unstable and therefore driven to decompose when in contact with metallic Li. One would expect some distribution of *x* values in the LBH‐LPSCl crystallites, since milling‐induced doping will not be homogeneous on an atomic scale. Therefore, borohydride incorporation will induce local variations in the thermodynamic stability of the lattice, and on the average may make the lattice even less thermodynamically stable. This further supports that argument that the enhanced electrochemical stability of the LBH‐ LPSCl is a kinetic effect due to a modified SEI.

It was observed that such disintegration of the PS_4_^3−^ units is induced by the reduction of P through electron transfer from the Li metal into the SSE at the interface. This process can be demonstrated by calculating the averaged charge of P, layer by layer, within the SSE parallel to the interface. The Bader charges of each ‘snapshot’ structure at specific simulation times from the AIMD data were used for this calculation. The obtained electron transfer across the interface against time for each interface model is shown in Figure 6c. It is found that the transfer (as measured by the color bar in the figure) from Li metal to P is limited in the BH_4_^−^‐containing model (*x* = 0.125), while there is severe electron transfer at the Li‐LPSCl interface. These results are further supported by the ion transfer across the interface. Due to the reduction reaction, the breaking‐up S from PS_4_^3−^ in the SSE will diffuse out of its orginal crystalline site (16*e* Wyckoff site) and move toward the interface layer to form detrimental interphases such as Li_2_S. Similar will happen for the S with the 4*c* Wyckoff site. Such a diffusion corresponds to the change of the S distribution in the structure, which can be characterized by the position deviation of the S anions along the *z*‐axis perpendicular to the interface,

(1) $$\sigma =\sqrt{\frac{1}{N}\sum_{i=1}^{N}{\left(z_{i}-\bar{z}\right)}^{2}},\;\bar{z}=\frac{1}{N}\;\sum_{i}^{N}z_{i}$$

The change of *σ* against the simulation time can measure the change of the S distributions over time. As shown in Figure 6d, it is found that there is severe S anion diffusion across the pristine Li‐LPSCl interface (black lines), while the Li‐Li_6_PS_5_(BH_4_)_0.125_Cl_0.875_ interface exhibit unchanged S distribution other than some fluctuations around a constant value over time (red lines). Both the electron and ion transfer results (Figure 6c,d) are consistent and coincide with the disintegration of PS_4_^3−^ units (Figure 6b) in the Li‐Li_6_PS_5_Cl interface model, suggesting that BH_4_^−^ is effective in passivating the interface against Li metal.

**Scheme 1:**
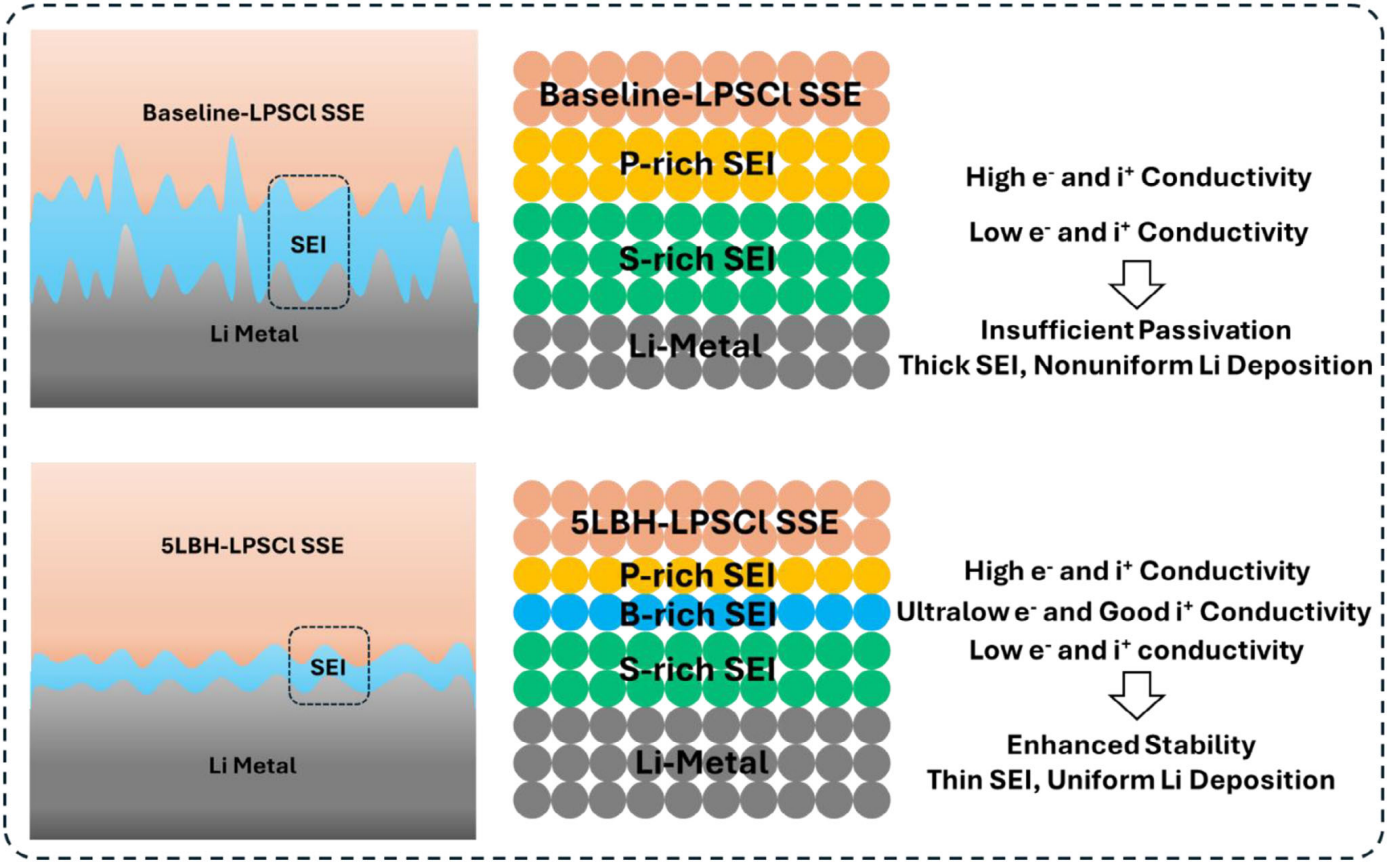
Illustration of the borohydride‐doping effect on the properties of Li‐SSE interphases.

**Scheme** **1** summarizes the effect of borohydride doping on the properties of Li‐SSE, comparing it to undoped baseline‐LPSCl. Since Li metal is thermodynamically unstable against LPSCl, an SEI forms upon contact and evolves with cycling. In baseline‐LPSCl, the SEI consists of a layered structure with P‐rich (predominately Li_3_P) and S‐rich (predominantly Li_2_S) phases. Li_2_S is a poor electronic conductor with a band gap of 3.39 eV and a poor ionic conductor (10^−7^ to 10^−11^ mS cm^−1^ in bulk and 10^−5^ to 10^−7^ mS cm^−1^ in thin‐film form).^[^ ^91^, ^92^ ^]^ By contrast, Li_3_P is both a good electronic (band gap of 0.70 eV) and ionic conductor (>0.1 mS cm^−1^).^[^ ^93^, ^94^ ^]^ As Li_3_P is adjacent to LPSCl SSE, any electron leakage through the surface layer leads to continuous LPSCl decomposition, thickening the SEI. Additionally, the poor ionic conductivity of Li_2_S causes an inhomogeneous Li‐ion flux across the interface, resulting in nonuniform Li electrodeposition/dissolution. For 5LBH‐LPSCl, borohydride doping introduces a B‐rich (predominately LiBH_4_) layer between the P‐rich and S‐rich layers in SEI. As LiBH_4_ is an electrical insulator with a large band gap of 6.81 eV, it acts as an electron barrier at the Li/SSE interface. This minimizes current leakage into Li_3_P and lowers the rate of LPSCl decomposition, thereby forming a thinner SEI. The thinner SEI in‐turn leads to enhanced ion transport and more uniform Li electrodeposition/dissolution. The phase LiBH_4_ exhibits relatively high ionic conductivity (>10^−5^ mS cm^−1^ in bulk and >0.1 mS cm^−1^ with confinement or doping).^[^ ^95^, ^96^, ^97^, ^98^ ^]^ Therefore, unlike Li_2_S, this layer does not inhibit ion transport. Consequently, borohydride‐doped LPSCl demonstrates improved SEI stability and superior electrochemical performance.

## 3 Conclusion

The interphase between lithium metal and argyrodite‐type Li_6_PS_5_Cl (LPSCl) plays a crucial role in the electrochemical stability of all solid‐state batteries (ASSBs) due to their inherent thermodynamic instability. Here it is demonstrated the mechanism by which borohydride doping into LPSCl significantly enhances its electrochemical stability. With 5 wt% LiBH_4_, the resulting 5LBH‐LPSCl SSE exhibits state‐of‐the‐art electrochemical performance. For example, symmetric cells with 5LBH‐LPSCl achieve a critical current density (CCD) of 7.3 mA cm^−2^, compared to 2.6 mA cm^−2^ in baseline cells. Furthermore, an ASSB employing lithium metal, 5LBH‐LPSCl SSE, and NMC811 cathode achieves stable cycling over 400 cycles at 0.5C with a capacity retention of 83%. An anode‐free ASSB (AF‐ASSB) with magnesium/tungsten coated copper (Mg/W‐Cu) collector is stable over 600 cycles, with capacity loss of only 0.04% per cycle.

In situ Li electrodeposition analysis was carried out utilizing X‐ray photoelectron spectroscopy (XPS) and time‐of‐flight secondary ion mass spectrometry (TOF‐SIMS). XPS results reveal an accelerated formation of metallic Li on the surface of BH_4_‐doped SSE, indicating the development of a faster kinetically stabilized SEI and improved Li wetting compared to baseline‐LPSCl. TOF‐SIMS analysis shows that the SEI consists of a layered structure with a P‐rich layer adjacent to the bulk SSE, and an S‐rich layer near the electrodeposited Li metal. Notably, borohydride doping introduces an additional B‐rich layer between the P‐rich and S‐rich layers, acting as an electron blocking barrier while allowing efficient ion transport. Theoretical modeling demonstrates that borohydride‐doping does not appreciably change the thermodynamics of LPSCl decomposition, namely there is no strong effect on the stability of PS_4_^3−^ units relative to Li metal.

## 4 Experimental Section

### Materials Preparation:—Argyrodite Li_6_PS_5_Cl (LPSCl) Solid‐State Electrolyte (SSE) Processing

Commercial Argyrodite Li_6_PS_5_Cl SSE was purchased from NEI Corporation, USA. Based on our previous work, a 24‐h wet ball milled LPSCl S SE exhibited the best performance in terms of critical current density (CCD) and cycling stability, therefore was chosen as the benchmark for current study. Briefly, four grams of LPSCl SSE were added to a 50 mL zirconia milling jar containing 20 zirconia milling balls (6 mm diameter). Then, 4.8 mL anhydrous m‐Xylene was added, and the milling jar was vacuum‐sealed inside the glovebox. Wet ball milling was conducted on a planetary ball mill machine (PM100, Retsch) at a speed of 300 rpm for 24 h with a 5‐min rest for every 20‐min milling. Finally, the electrolyte was obtained after drying in the glove box at 80 °C overnight. Unless otherwise noted, the resulting SSE was referred to as “baseline‐LPSCl” sample in this study.

To prepare LiBH_4_‐doped LPSCl, two hundred milligrams (5 wt%) of LiBH_4_ (95%, BTC, USA) were mixed with 4 grams of LPSCl SSE, followed by a similar wet ball milling process. The resultant was denoted as 5LBH‐LPSCl. For comparison, samples with a smaller amount (40 mg, 1 wt%) and a higher amount (400 mg, 10 wt%) of LiBH_4_ were also prepared and denoted as 1LBH‐LPSCl and 10LBH‐LPSCl, respectively. Other additives including LiBF_4_, LiPF_6_, and LiBO_2_ were also prepared using a similar approach with a fixed amount of 200 mg (5 wt%). The resulting specimens were named as 5LBF‐LPSCl, 5LPF‐LPSCl, and 5LBO‐LPSCl, respectively. To prepare the hand‐ground LiBH_4_‐LPSCl sample, 200 mg of LiBH_4_ (5 wt%) was mixed with 4 g of 24‐h ball milled baseline‐LPSCl SSE. The mixture was then hand‐ground in a mortar for 10 min. The resulting sample was labeled as 5LBH/LPSCl.

### Cell Assembly

All solid‐state symmetric Li/SSE/Li cells: An amount of 150 mg of baseline or modified SSE powder was first pressed at 530 MPa for 1 min in a polyether ether ketone (PEEK) mold with a diameter of 12 mm, employing a hydraulic press (YLJ‐24TS, MTI Corporation). To obtain Li foils, a cleaned Li chip (GoodFellow, USA) was sandwiched in a plastic bag and rolled to a thickness of ≈100 µm. This was performed inside an Ar atmosphere glove box. For Li/SSE/Li cells, eight‐millimeter diameter Li electrodes were punched out and attached to either side of the compact without being excessively compressed. Care was taken to prevent their mechanical deformation during this process. Afterward, the cell was positioned in the cell case and tightened using a torque wrench. A torque of 20 lb‐in (corresponding to 9 MPa pressure) was employed, the pressure being measured using a torque wrench with additional torque‐pressure calibration being performed at Oak Ridge National Laboratory.

All solid‐state symmetric Ni/SSE/Ni cells: An amount of 150 mg of baseline or modified SSE powder was added to the PEEK, followed by attaching Ni foils with a diameter of 12 mm to each side of the composite and pressing at 530 MPa for 1 min. Afterward, the cell was positioned in the cell case and tightened to a final torque of 20 lb‐in. The Ni/SSE/Ni cells were employed for electrical conductivity and Coulometric titration time analysis.

All solid‐state symmetric Li/SSE/Cu and Li/SSE/SS cells: For the asymmetric half‐cells, 150 mg of baseline or modified SSE powder was first pressed at 50 MPa using the same PEEK molds. A 12 mm diameter disk of copper (Cu) or stainless steel (SS) foil was attached to one side of the compact and pressed at 530 MPa for 1 min. Next an 8 mm diameter Li disk was attached to the other side of the compact without external pressure. The cell was then positioned in the cell case and tightened to a final torque of 20 lb‐in, the same pressure used for symmetric cell testing.

**All solid‐state Li/SSE/NMC full cells**: The composite cathode was prepared by hand mixing LiNbO_3_‐coated NMC811 cathode (NEI Corporation, USA) with raw SSE powder and carbon nanofiber (VGCF, Sigma Aldrich) at a weight ratio of 7:3:0.3 in a mortar for 40 min. When assembling the battery, 150 mg baseline or modified LPSCl SSE powder was first pressed under 50 MPa in a PEEK mold. Then the composite cathode powder was uniformly dispersed on one side of the SSE pellet and the areal capacity of cathode was ≈1.5 mAh cm^−2^ for typical measurements. A piece of aluminum foil was inserted between the composite cathode and the titanium plunger. The entire cell was further pressed under 867 MPa for 2 mins to obtain an electrolyte pellet in close contact with the cathode layer. Then an 8 mm diameter Li foil was attached to the other side of the electrolyte pellet without being compressed. The laminated battery was finally positioned in the cell case and tightened to a final torque of 20 lb‐in.

Anode‐free all solid‐state full cells: 150 mg 5LBH‐LPSCl SSE powder was first pressed under 50 MPa in a PEEK mold. Then the composite cathode powder was uniformly dispersed on one side of the SSE pellet, while a current collector (Mg/W‐Cu), prepared according to our previous work,^[^ ^35^ ^]^ was placed on the opposite side. The areal capacity of cathode was ≈1.5 mAh cm^−2^ for typical measurements and ≈6 mAh cm^−2^ for high mass‐loading experiments. A piece of aluminum foil was inserted between the composite cathode and the titanium plunger. The entire cell was then pressed under 867 MPa for 2 mins, followed by placement into the cell case and tightening to a final torque of 30 lb‐in (≈14 MPa).

### Electrochemical Measurements

Galvanostatic cycling was conducted on Land CT2001A battery testers. The current densities and capacities employed during testing are noted in the descriptions of the analyses and the captions of the associated figures. Electrochemical impedance spectroscopy (EIS) tests were recorded on a Gamry electrochemical workstation in the frequency range of 1 MHz – 1 Hz with an amplitude of 10 mV. Ionic conductivity of SSEs was calculated based on the equation of σ = L/(R_b_A), where L is the thickness of the electrolyte pellet, R_b_ is the bulk resistance of SSE, and A is the area of the SSE layer. Electrical conductivity measurements of SSEs were performed using Ni/SSE/Ni symmetric cells and a constant voltage was maintained at 0.5 V for 2 h. The ohmic resistance (R) was determined using the equation R = U/I, where U is the applied voltage, and I is the equilibrium current. The Coulometric titration time analysis (CTTA) was performed in Li/SSE/SS asymmetric cells followed by a previous study with slight modification. Briefly, in each titration step a current of 0.01 mA cm^−2^ was applied for 10 min. In the next step, the cell is kept in the open‐circuit voltage (OCV) state until the cell voltage reaches a cut‐off limit of 0.05 V, after which the next titration step was immediately performed. These procedures were repeated as many times as necessary (≈250 h in this study). All electrochemical measurements in this work were carried out at room temperature (≈23 °C), unless otherwise specified.

### Material Characterization

Room‐temperature and cryogenic scanning electron microscopy (SEM) and focused‐ion beam (FIB) SEM were both performed on a Thermo Scientific Scios 2 Dual Beam SEM/FIB with a Leica VCT cryogenic stage and energy dispersive X‐ray spectrometer (EDXS). To preserve the structural integrity of the beam‐sensitive Li‐based materials and to reduce artificial inclusion, the sample was cooled to −150 °C. The Ga^+^ FIB milling was performed at an accelerating voltage of 30 keV. Transmission electron microscopy (TEM) lamella sample preparation was performed on a Scios 2 Dual Beam SEM/FIB. The sample was cooled to −150 °C and Ga^+^ FIB milling was performed with an accelerating voltage of 30 keV. Final thinning was carried out using a beam current of 50 pA. Scanning transmission electron microscopy (STEM) was performed using a Titan ETEM operating at 300 keV. Electron energy loss spectroscopy (EELS) was carried out using a Gatan Biocontinuum/K3‐IS. X‐ray diffraction (XRD) profiles were recorded on Rigaku Miniflex 600 diffractometer with Cu Kα radiation (λ = 1.54178 Å) at a scan rate of 5° per minute within the 2θ range from 10° to 80°. X‐ray photoelectron spectroscopy (XPS) analyses were performed using a Kratos Axis Ultra DLD XPS (manufactured by Kratos Analytical, Inc.), equipped with an Al K𝛼 monochromatic X‐ray source with a power set at 120 W. The photoelectrons were collected with an emission angle (EA) of 90° and from a sample area of 300 µm × 700 µm. For high‐resolution spectra, the measurements were performed in constant‐analyzer‐energy (CAE) mode with a pass energy of 20 eV and a step size of 0.1 eV (full‐width‐at‐half‐maximum of the peak for Ag 3d_5/2_ is 0.77 eV). The residual pressure in the analytical chamber was ≈5 × 10^−9^ Torr. The acquired spectra were fitted using CasaXPS software.

### In Situ XPS Li Electrodeposition

A total of 105 mg of SSE was added to a 10‐mm pellet die and pressed at 50 MPa for 1 min. An 8‐mm Li foil was then gently pressed onto one side of the pellet, which was subsequently placed in a PEEK cell mold and tightened to a pressure of 9 MPa. The pellet was mounted onto an XPS holder with the Li metal facing a carbon tape and transferred from the glovebox to the XPS chamber using an air‐tight transfer vessel to prevent air exposure. Prior to in situ Li electrodeposition, the sample surface was cleaned with an Ar cluster sputter gun operated at a voltage of 2.5 keV for 4 min. This procedure effectively removed adventitious hydrocarbons without causing damage to the sample. During the in situ Li electrodeposition, a subsidiary electron neutralizer was employed to expose the sample surface to a low‐energy electron beam with a fixed beam current of 5 µA. Both the Ar^+^ neutralizer and X‐ray source were active during this process. High‐resolution spectra were collected after reaching a specific charge, with both two neutralizers turned off during the data acquisition.

### In Situ TOF‐SIMS Li Electrodeposition

The sample preparation procedures are identical to the abovementioned and transferred to the TOF‐SIMS analysis chamber from the glovebox using an air‐tight transfer vessel. The TOF‐SIMS depth profiling of the lithium/SSE interface was investigated using the latest TOF‐SIMS instrument from ionTOF GmbH, the M6 (Germany, 2023). The Nanoprobe 50 analysis ion gun uses a Bi^+^ source shooting 20 ns pulses at 30 keV ion energy and ≈0.3 pA measured sample current. For surface ablation a Cs^+^ (500 eV ion energy, ≈40 nA measured sample current) ion beam was employed with raster scanning areas of 300 µm × 300 µm. The analysis ion beam raster scans areas of 100 µm × 100 µm centered within the Cs^+^ sputtered areas. The depth profiles were acquired in noninterlaced mode, that is, sequential sputtering and analysis. All detected ions had negative polarity with a >8000 mass resolution. The measurements were performed in UHV at ≈5 × 10^−10^ mbar base pressure. The electrodeposited Li overlayer was grown with a continuous electron beam (21 eV, ≈25 µA sample current) sputtering the solid electrolyte surface for ≈3 h. The analysis areas toward the edges of the e‐beam sputtered regions were selected to effectively control the thickness of the Li overlayer, thereby optimizing the data acquisition time.

### Solid‐State NMR Spectroscopy

Solid‐state NMR experiments were carried out using an Agilent DD2 400 MHz NMR spectrometer equipped with a 3.2 mm Chemagnetics magic‐angle spinning (MAS) probe. Samples were packed into the MAS rotors in an Ar glovebox and nitrogen gas was used to spin the samples to 10 kHz, to minimize oxygen exposure. All ^7^Li, ^11^B, and ^31^P NMR spectra were acquired using a Bloch decay sequence, using 2.0, 4.5, and 2.0 90° excitation pulses, respectively. Relaxation delays were set to 0.25, 2.0 and 16 s for ^7^Li, ^11^B, and ^31^P, respectively, and 2, 64, and 64 scans were accumulated.

^11^B{^31^P} REDOR was performed using a Bruker AVANCE NEO 600 MHz NMR spectrometer equipped with a triple‐resonance PhoenixNMR 1.3 mm fast‐MAS probe. ^11^B Excitation and refocusing pulses lasted 7.5 and 15 µs, respectively, while the ^31^P REDOR pulses lasted 5 µs. The experiment was carried out with a 10 kHz MAS frequency and 256 scans were acquired for each sub‐spectrum with a 2 s recycle delay.

### Computational Method

Density functional theory (DFT) calculations with Perdew‐Burke‐Ernzerhof (PBE) generalized gradient approximation (GGA)^[^ ^99^ ^]^ implemented in the VASP package^[^ ^100^ ^]^ were carried to optimize the built interface structures. The projector augmented wave (PAW) pseudopotential method^[^ ^101^ ^]^ was used. A dense Monkhorst‐Pack **k**‐point mesh with lattice parameters >12 Å was adopted in the simulations with a cutoff energy of 500 eV. The energy and force convergences were set to 10^−6^ eV and 0.01 eV Å^−1^, respectively. Since the doping (molar) concentration of BH_4_^−^ is estimated to be less than 55 mol%, we built the SSE model Li_6_PS_5_(BH_4_)_x_Cl_1−x_ (*x* = 0 to 0.625 with a step of 0.125 or 12.5%) compared to the pristine Li_6_PS_5_Cl. The interface model between the SSE and the Li‐metal is built as coherent heterointerfaces in a Li metal‐SSE‐Li metal symmetric configuration, allowing the SSE and Li supper‐lattices to form periodicities along the two interfacial planes. The SSE and Li metal are both cleaved and matched at the (100) surfaces, since it has been found that the (100) surface of Li_6_PS_5_Cl coupled with the (100) surface of Li metal entails the highest interfacial stability.^[^ ^102^ ^]^ The initial distance between the SSE and Li metal labs are set to 60% of the sum of the van der Waals radii of the terminal elements in close contact. The built Li‐SSE interface models show small mismatch stresses less than 1 kbar (0.1 GPa). The models were fully optimized and subject to ab initio molecular dynamics (AIMD) simulations at room temperature (300 K). Each AIMD simulation was performed on the N*V*T ensemble with a time step of 1 fs. 20 ps of simulation for each system was allowed for the system to reach thermal equilibrium before collecting data for another 200 ps for statistical analysis.

## Conflict of Interest

The authors declare no conflict of interest.

## Supporting information



Supporting Information

## Data Availability

The data that support the findings of this study are available from the corresponding author upon reasonable request.
