# Supplementary material for: Understanding the Role of Borohydride Doping in Electrochemical Stability of Argyrodite Li6PS5Cl Solid‐State Electrolyte
Source: Adv Mater. 2025 Jul 15;37(40):2506095. doi: 10.1002/adma.202506095 (PMC12510284; doi:10.1002/adma.202506095)
Supplement: Supplementary file 1 — Supporting Information [file ADMA-37-2506095-s001.docx]

**Understanding the Role of Borohydride Doping in Electrochemical Stability of Argyrodite Li_6_PS_5_Cl Solid-State Electrolyte**

Yixian Wang^1*^, Vikalp Raj^1^, Qianqian Yan^2^, Cole D. Fincher^2^, Yuanshun Li^3^, Rohit Raj^1^, Hugo Celio^1^, Andrei Dolocan^1^, Guang Yang^3^, Frédéric A. Perras^4,5^, Yet-Ming Chiang^2^, John Watt^6^, Hong Fang^7,8^, Puru Jena^9^, David Mitlin^1*^

*yixwang@utexas.edu, *david.mitlin2@utexas.edu

^1^Materials Science and Engineering Program, Walker Department of Mechanical Engineering and Texas Materials Institute, The University of Texas at Austin, Austin, TX 78712, USA

^2^Department of Materials Science & Engineering, Massachusetts Institute of Technology, Cambridge, MA 02139, USA

^3^Chemical Sciences Division, Oak Ridge National Laboratory, Oak Ridge, TN 37830, USA

^4^Chemical and Biological Sciences Division, Ames National Laboratory, Ames, IA 50011, USA

^5^Department of Chemistry, Iowa State University, Ames, IA 50011, USA

^6^Center for Integrated Nanotechnologies, Los Alamos National Laboratory, Los Alamos, NM 87545, USA

^7^Department of Physics, Rutgers University, Camden, NJ 08102, USA

^8^Center for Computational and Integrative Biology, Rutgers University, Camden, NJ 08103, USA

^9^Department of Physics, Virginia Commonwealth University, Richmond, VA 23238, USA

**
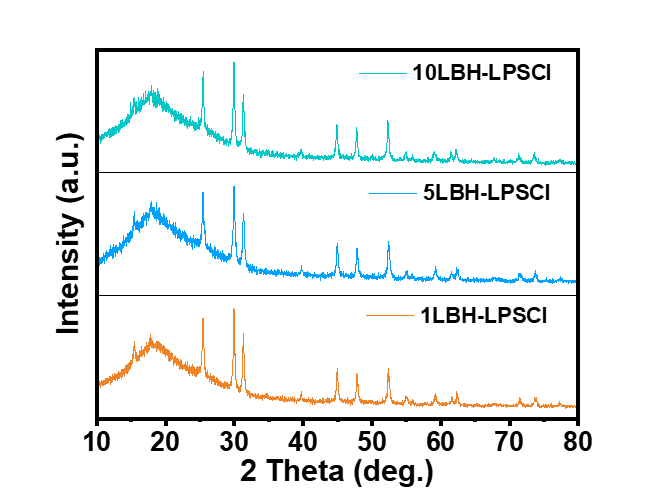
**

**Figure S1.** XRD profiles of LBH-doped LPSCl SSEs.

**
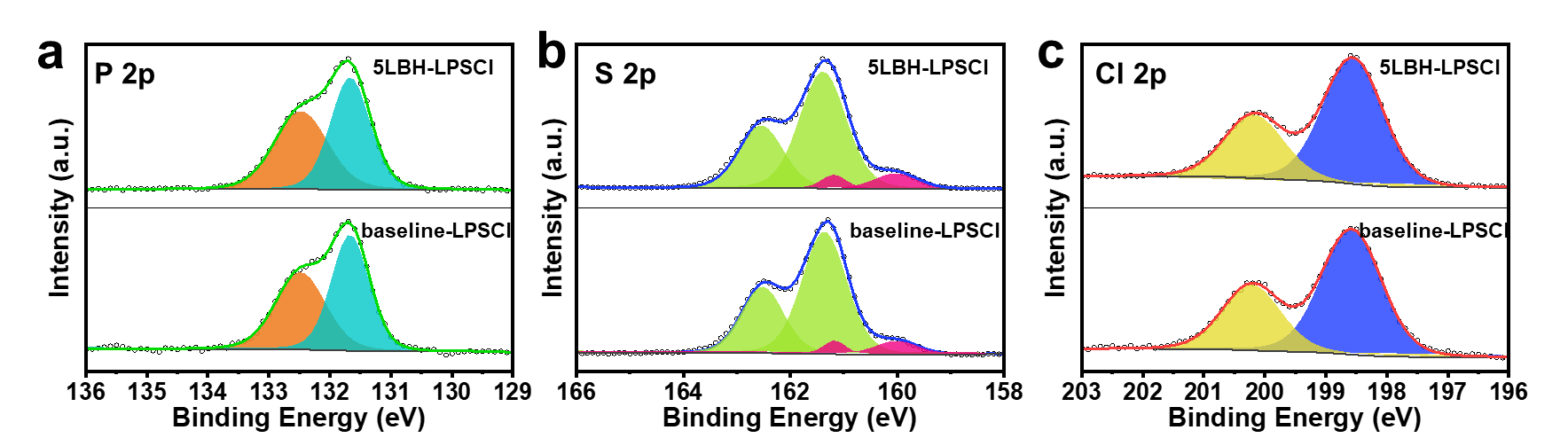
**

**Figure S2.** High-resolution XPS **(a)** P 2p, **(b)** S2p, and **(c)** Cl 2p spectra of 5LBH-LPSCl and baseline-LPSCl SSEs.

**
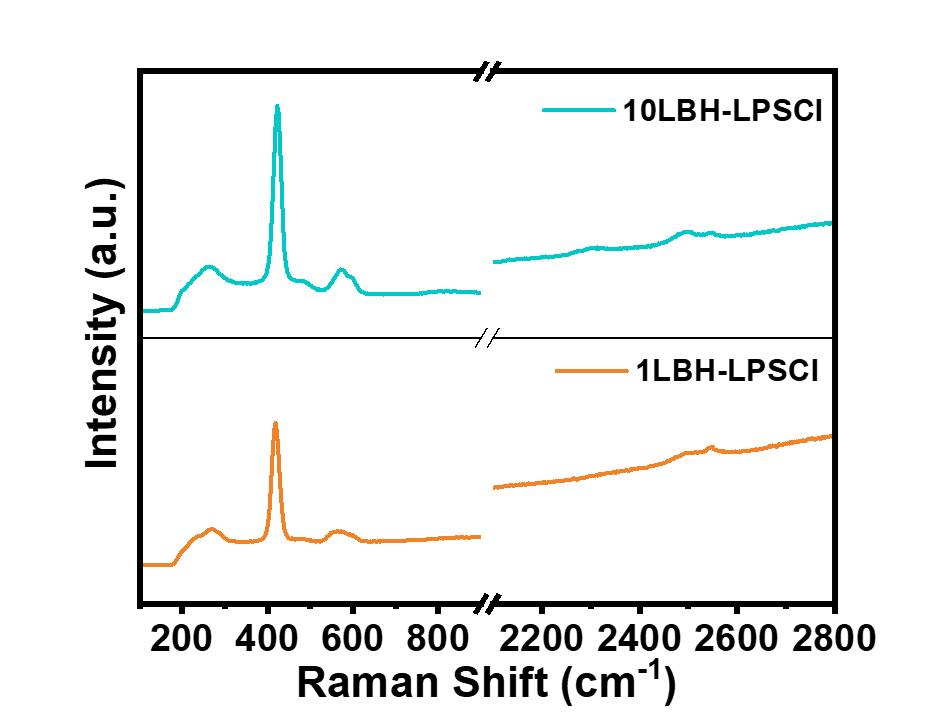
**

**Figure S3.** Raman spectra of 1LBH-LPSCl and 10LBH-LPSCl SSEs.

**
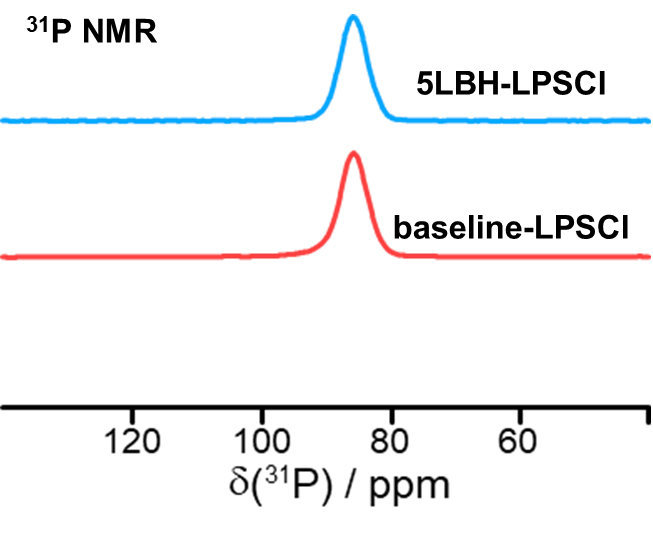
**

**Figure S4.** Solid-state ^31^P NMR spectra of 5LBH-LPSCl and baseline-LPSCl SSEs.

**
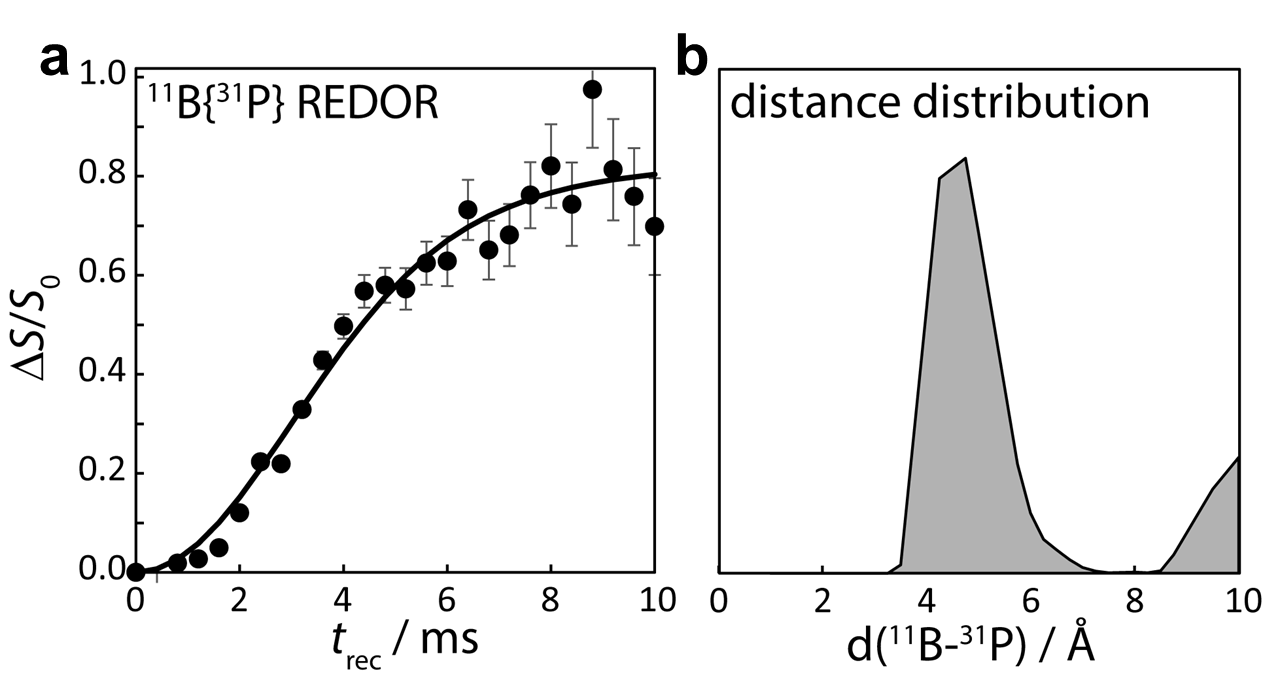
**

**Figure S5. (a)** ^11^B{^31^P} REDOR dephasing curve and fit. **(b)** Distance distribution from the REDOR transform.

**
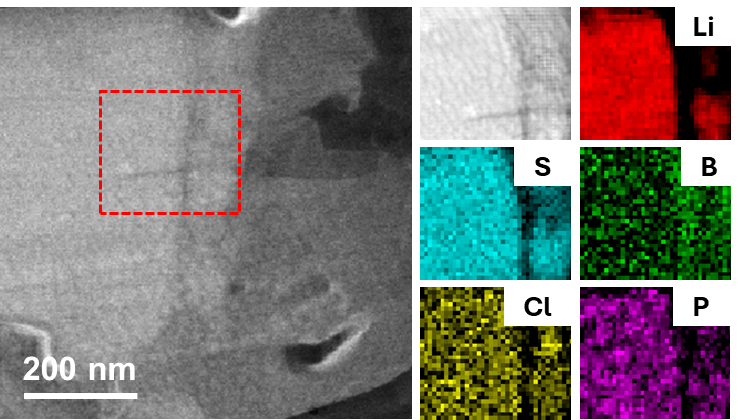
**

**Figure S6.** Cryo-TEM EELS analysis of 5LBH-LPSCl.

**
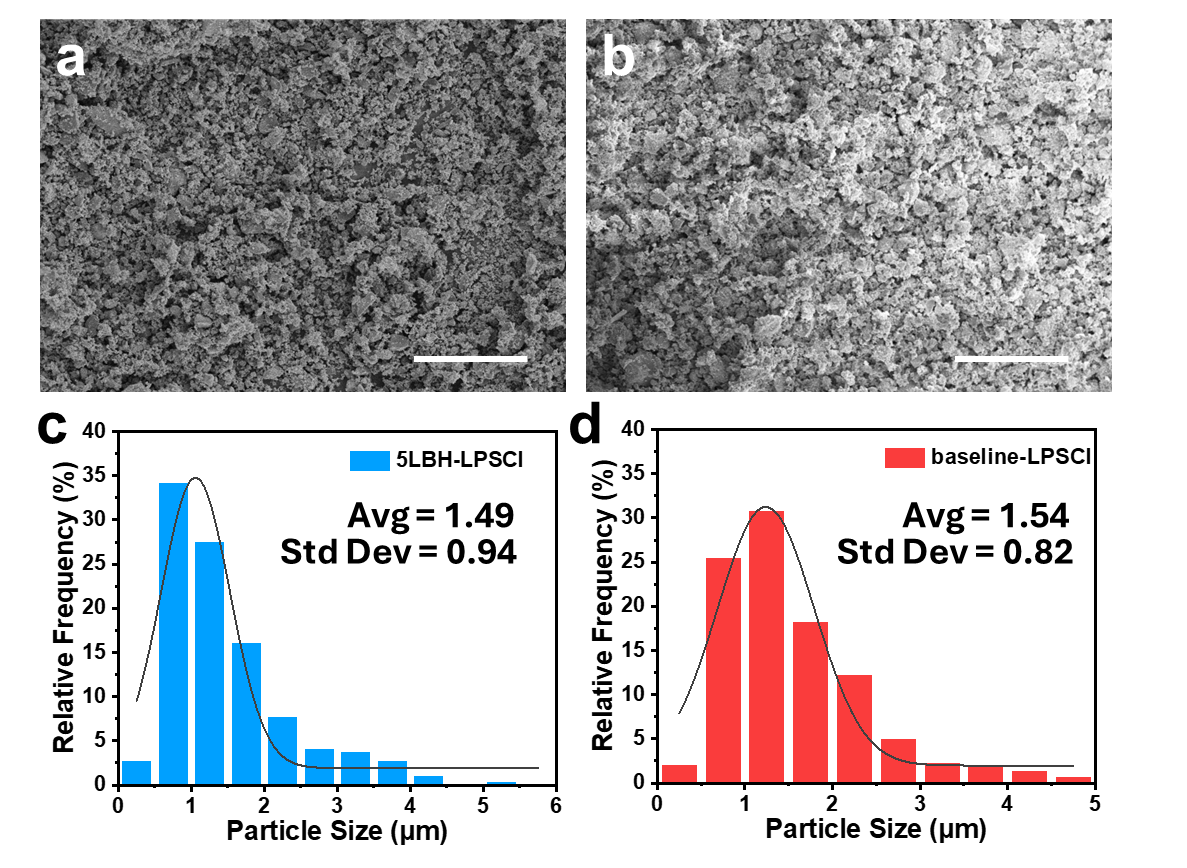
**

**Figure S7. (a, b)** SEM images and **(c, d)** particle size distribution profiles of 5LBH-LPSCl and baseline-LPSCl SSEs. The scale bar is 50 μm.


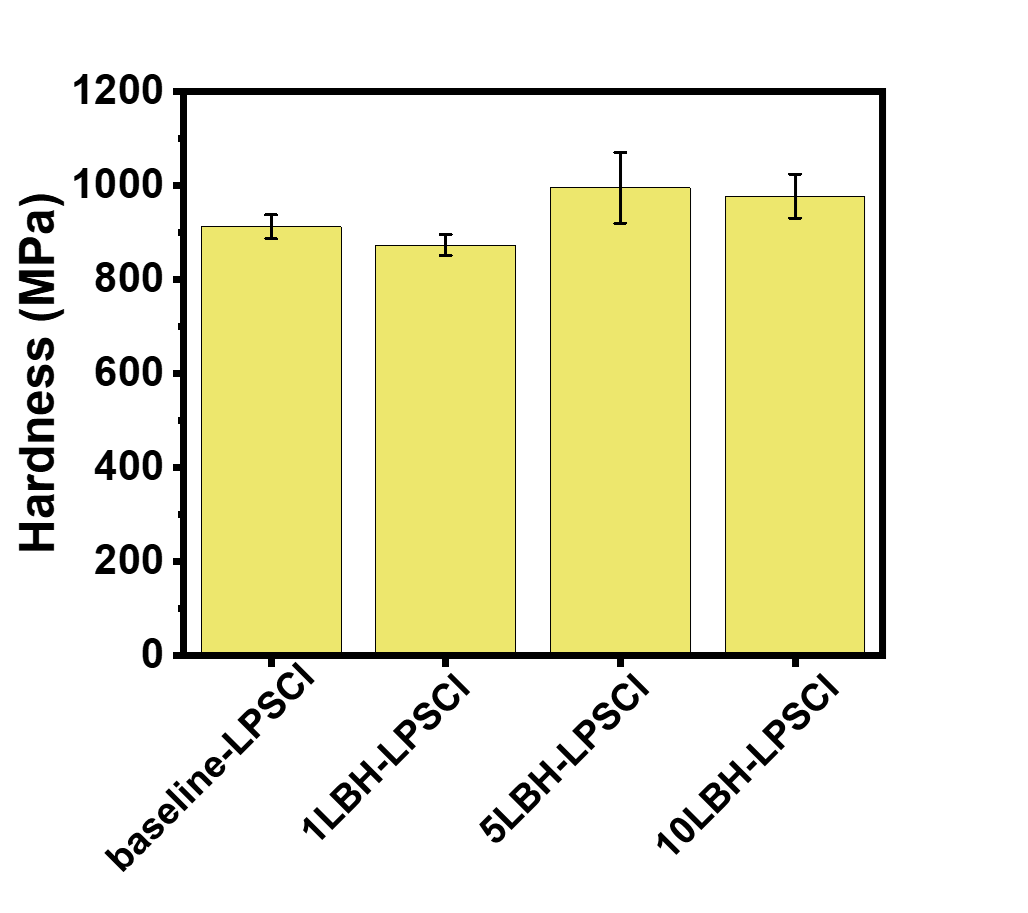


**Figure S8.** Hardness measurement of the LBH-doped and baseline-LPSCl SSEs.


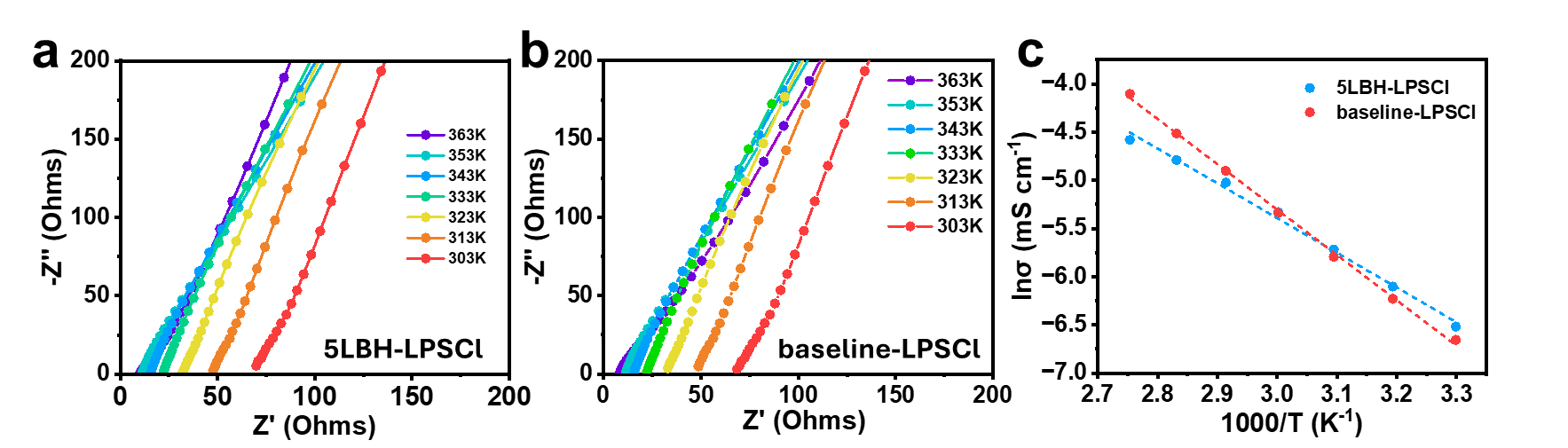


**Figure S9.** Temperature-dependent Nyquist plots of **(a)** 5LBH-LPSCl and **(b)** baseline-LPSCl cells employing Ti blocking electrodes. **(c)** Arrhenius plot of 5LBH-LPSCl and baseline-LPSCl.


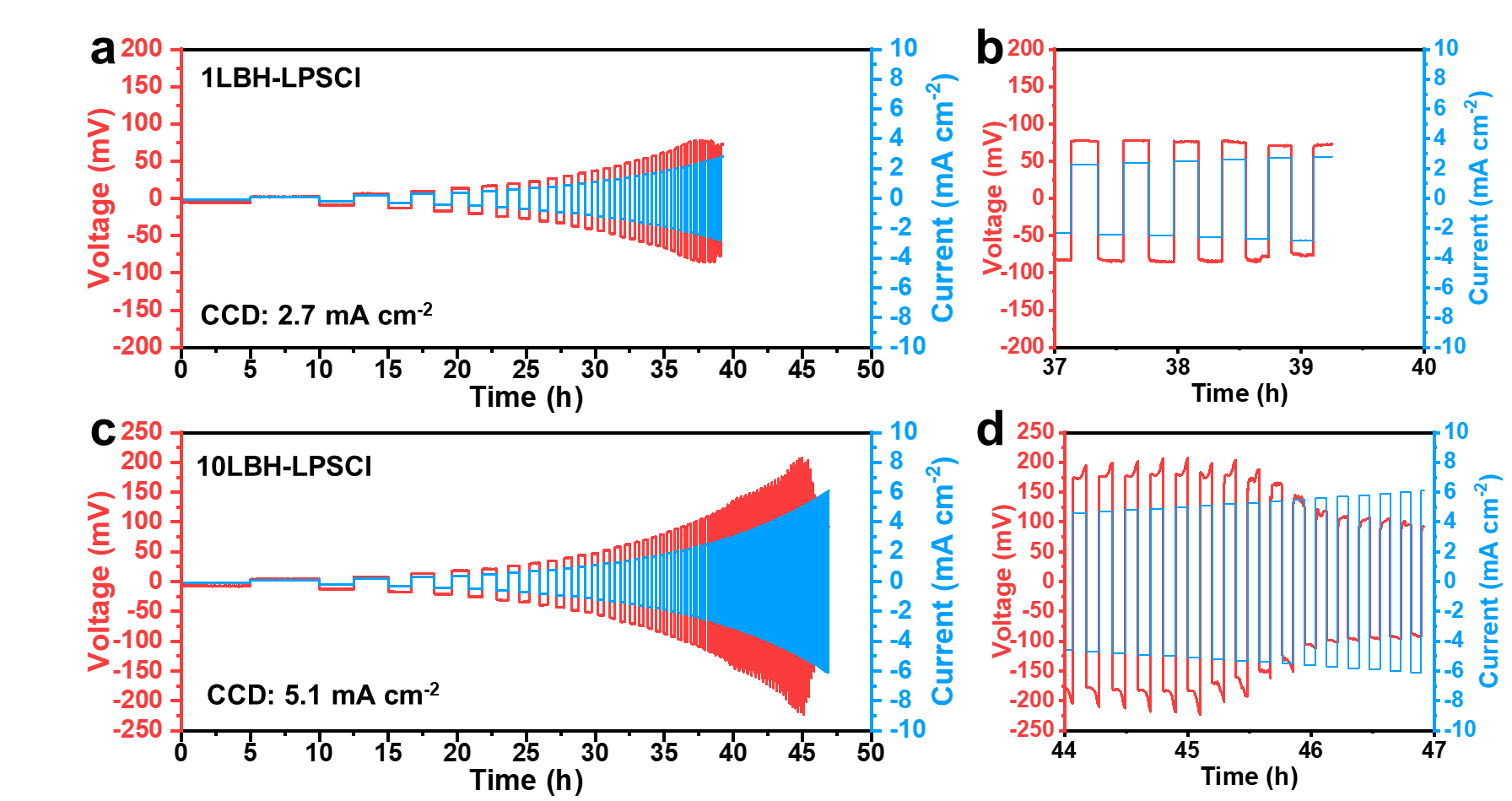


**Figure S10. (a, c)** CCD tests and **(b, d)** representative galvanostatic profiles of Li symmetric cells using **(a, b)** 1LBH-LPSCl and **(c, d)** 10LBH-LPSCl SSEs.


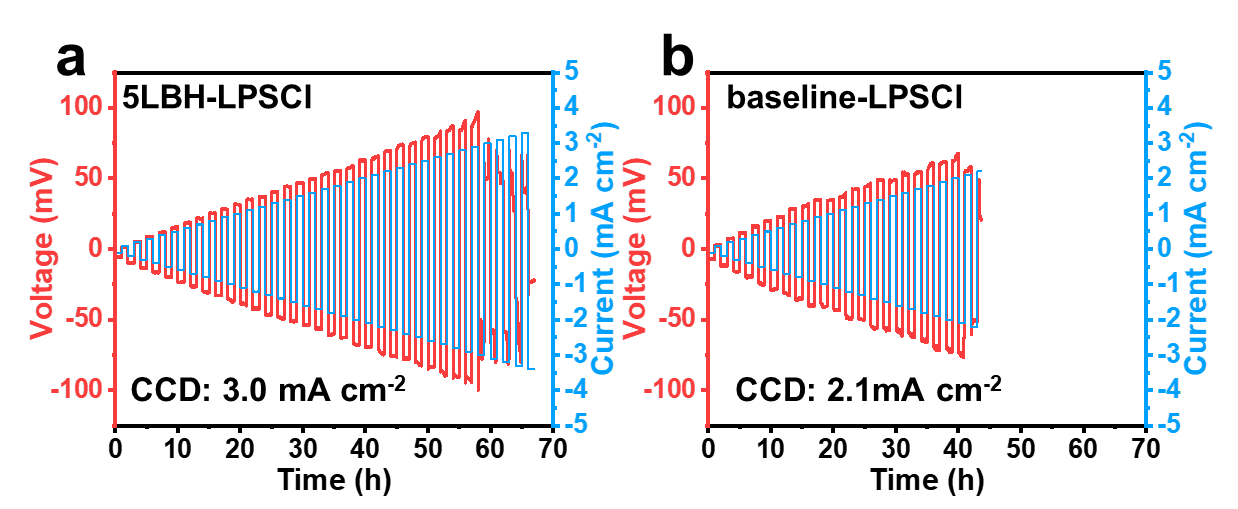


**Figure S11.** Constant time CCD tests of Li symmetric cells using **(a) 5**LBH-LPSCl and **(b)** baseline-LPSCl SSEs.


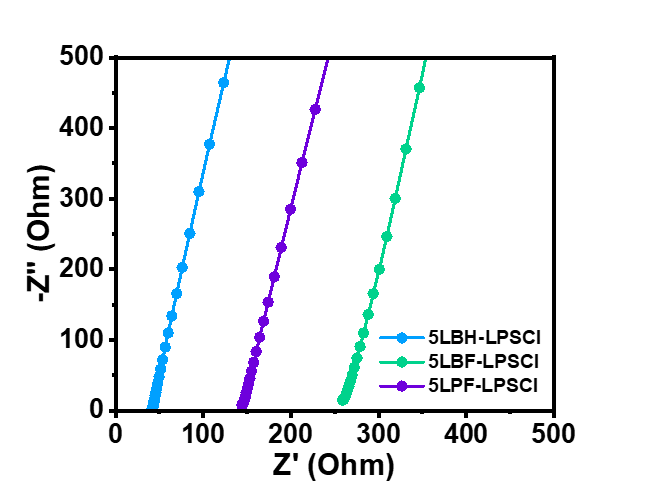


**Figure S12.** Nyquist plots of 5LBH-LPSCl, 5LBF-LPSCl, and 5LPF-LPSCl SSEs.


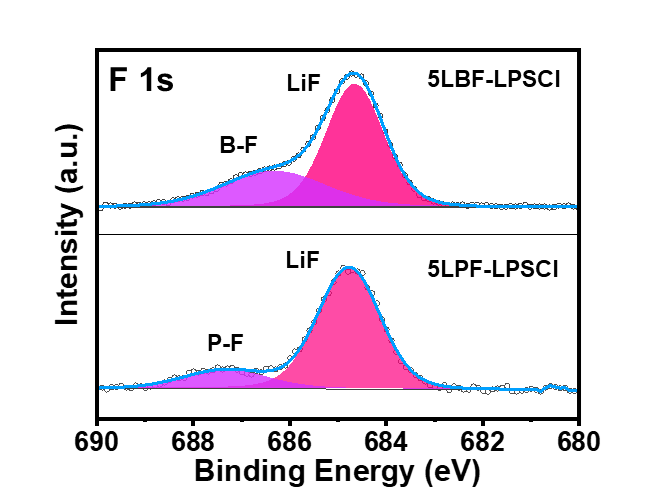


**Figure S13.** High-resolution F 1s spectra of 5LBF-LPSCl and 5LPF-LPSCl SSEs.


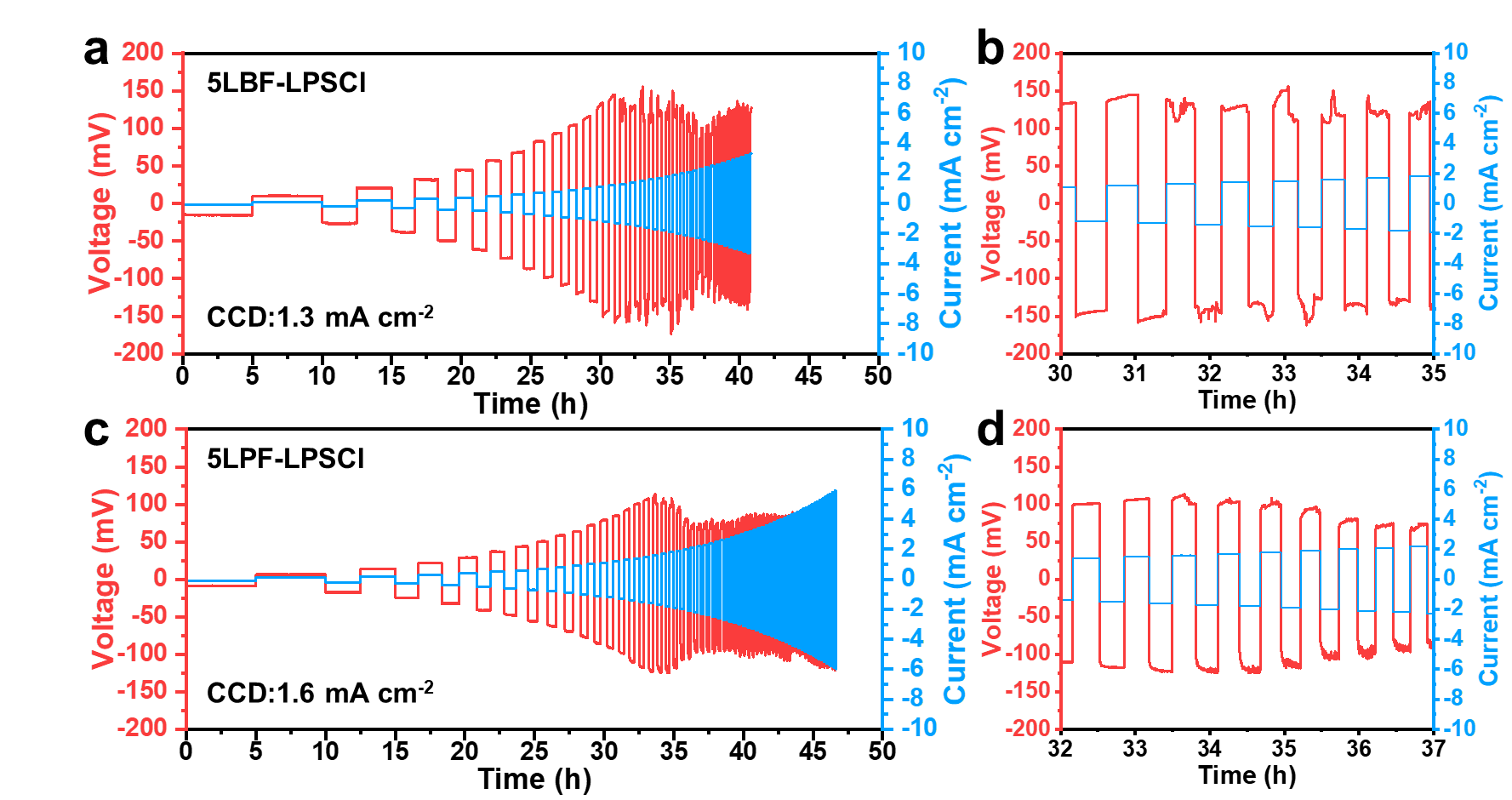


**Figure S14. (a, c)** CCD tests and **(b, d)** representative galvanostatic profiles of Li symmetric cells using **(a, b)** 5LBF-LPSCl and **(c, d)** 5LPF-LPSCl SSEs.


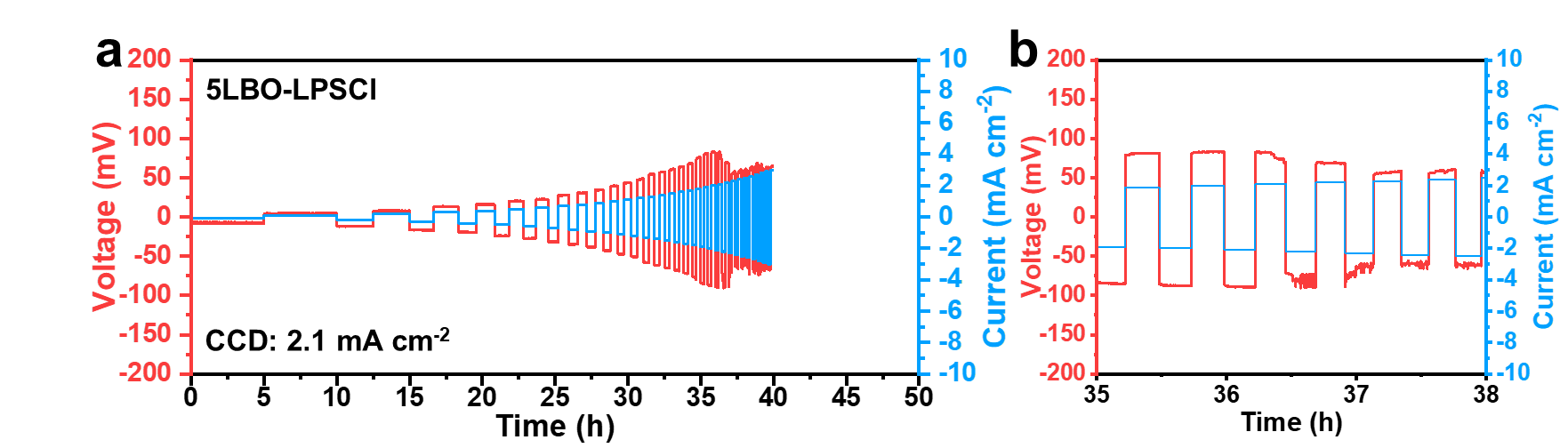


**Figure S15. (a)** CCD tests and **(b)** representative galvanostatic profile of Li symmetric cells using 5LBO-LPSCl SSE.


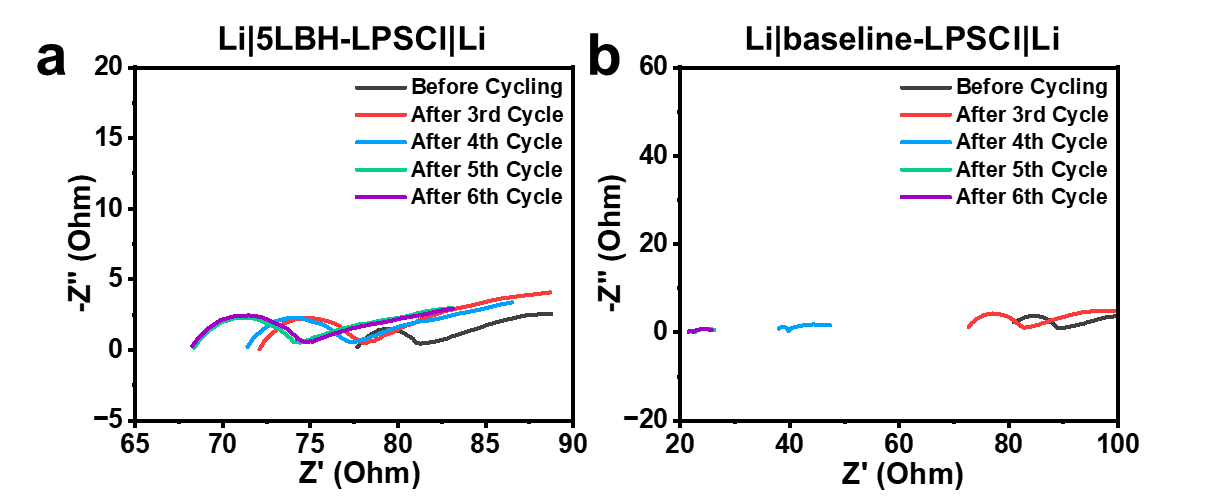


**Figure S16.** Nyquist plots of Li|5LBH-LPSCl|Li and Li|baseline-LPSCl|Li symmetric cells, tested at 0.5 mA cm^−2^/1 mAh cm^−2^ for 3 cycles, followed by 2 mA cm^−2^/1 mAh cm^−2^ for the remainder.


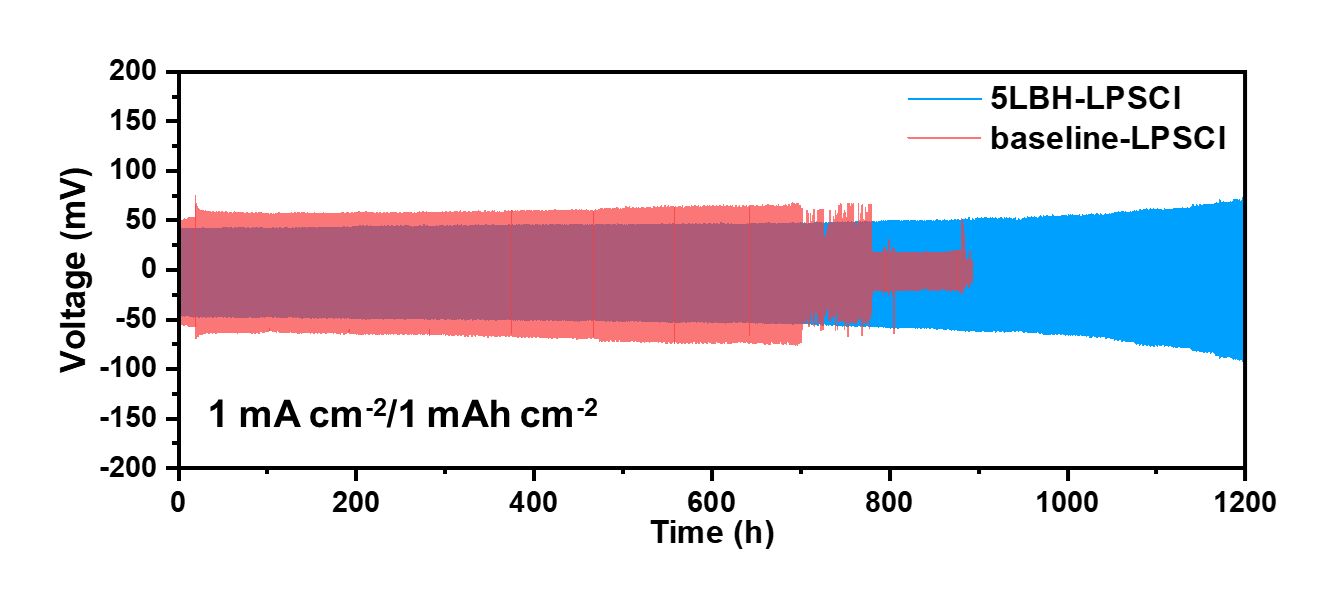


**Figure S17.** Cycling performance of symmetric Li cells using 5LBH-LPSCl and baseline-LPSCl SSEs, tested at 1 mA cm^-2^ to 1 mAh cm^-2^.


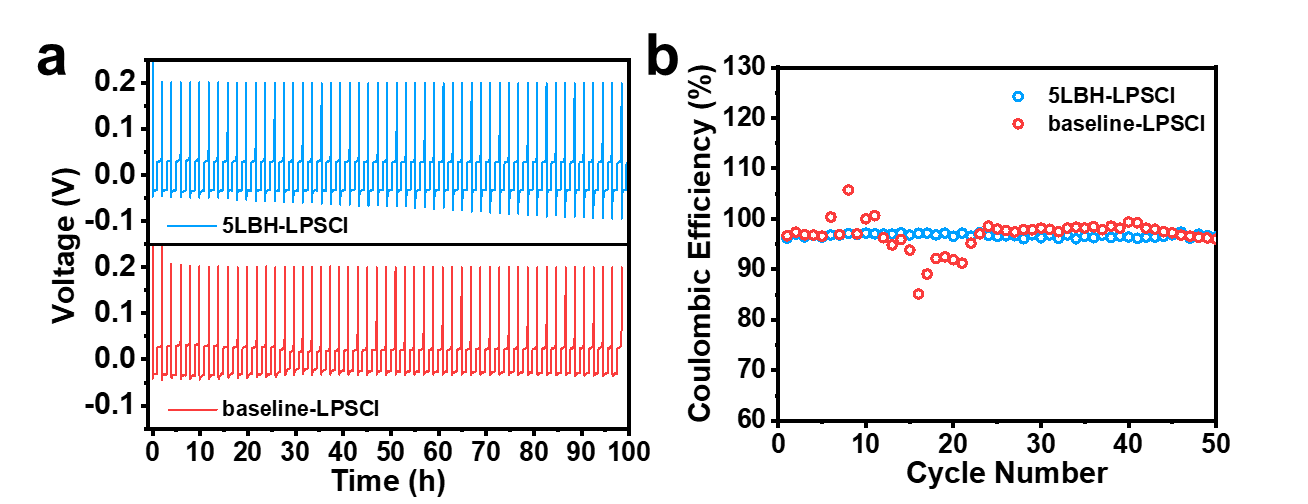


**Figure S18. (a)** Galvanostatic cycling profiles and **(b)** cycling CEs of Li|5LBH-LPSCl|Cu and Li|baseline-LPSCl|Cu cells.


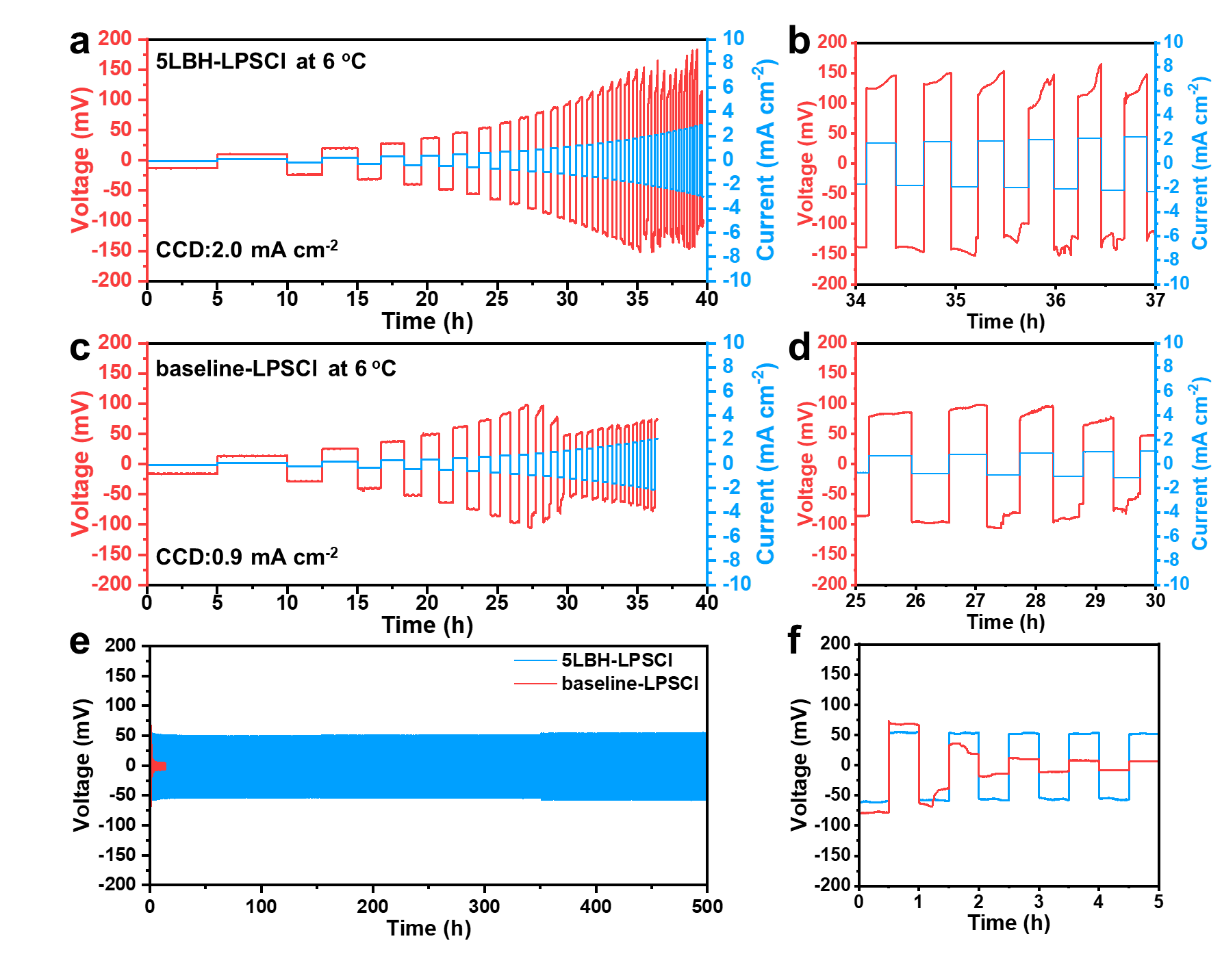


**Figure S19.** Low-temperature (6 ^o^C) **(a, c)** CCD tests and **(b, d)** representative galvanostatic profiles of Li symmetric cells using **(a, b)** 5LBH-LPSCl and **(c, d)** baseline-LPSCl SSEs. **(e)** Galvanostatic cycling performance tested at 0.5 mA cm^−2^/0.25 mAh cm^−2^. **(f)** Representative galvanostatic profiles from 1^st^ to 5^th^ cycles.


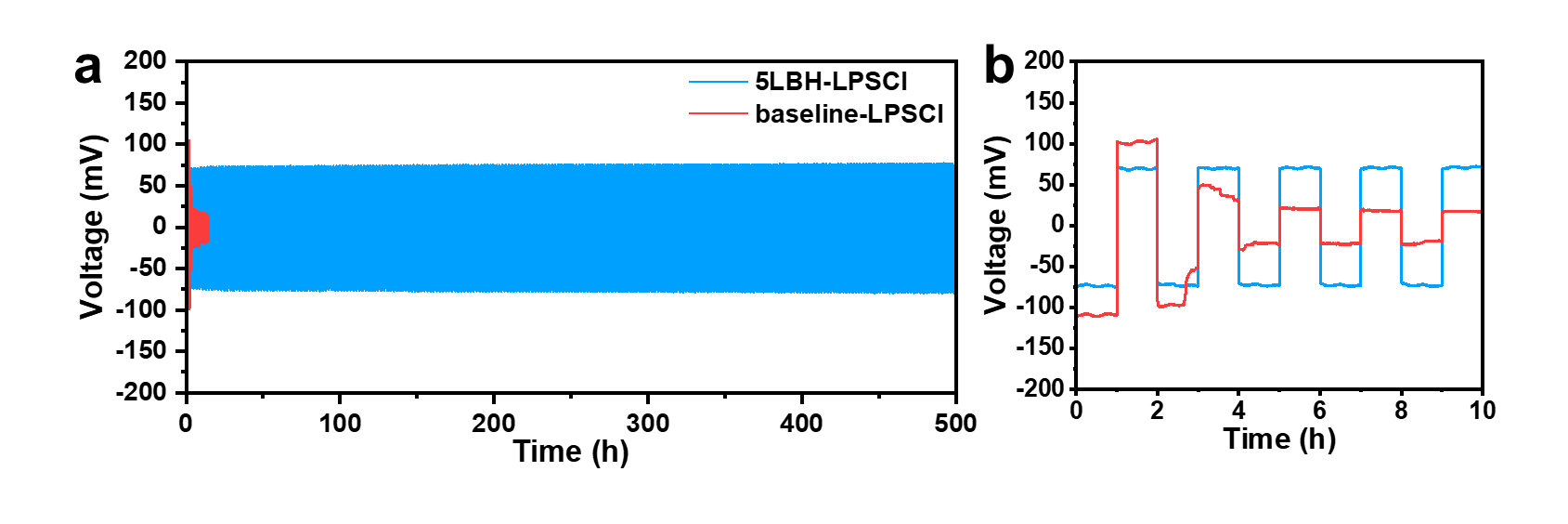


**Figure S20.** Ultralow-temperature (-14 ^o^C) **(a)** Galvanostatic cycling performance tested at 0.2 mA cm^−2^/0.2 mAh cm^−2^. **(b)** Representative galvanostatic profiles from 1^st^ to 5^th^ cycles.


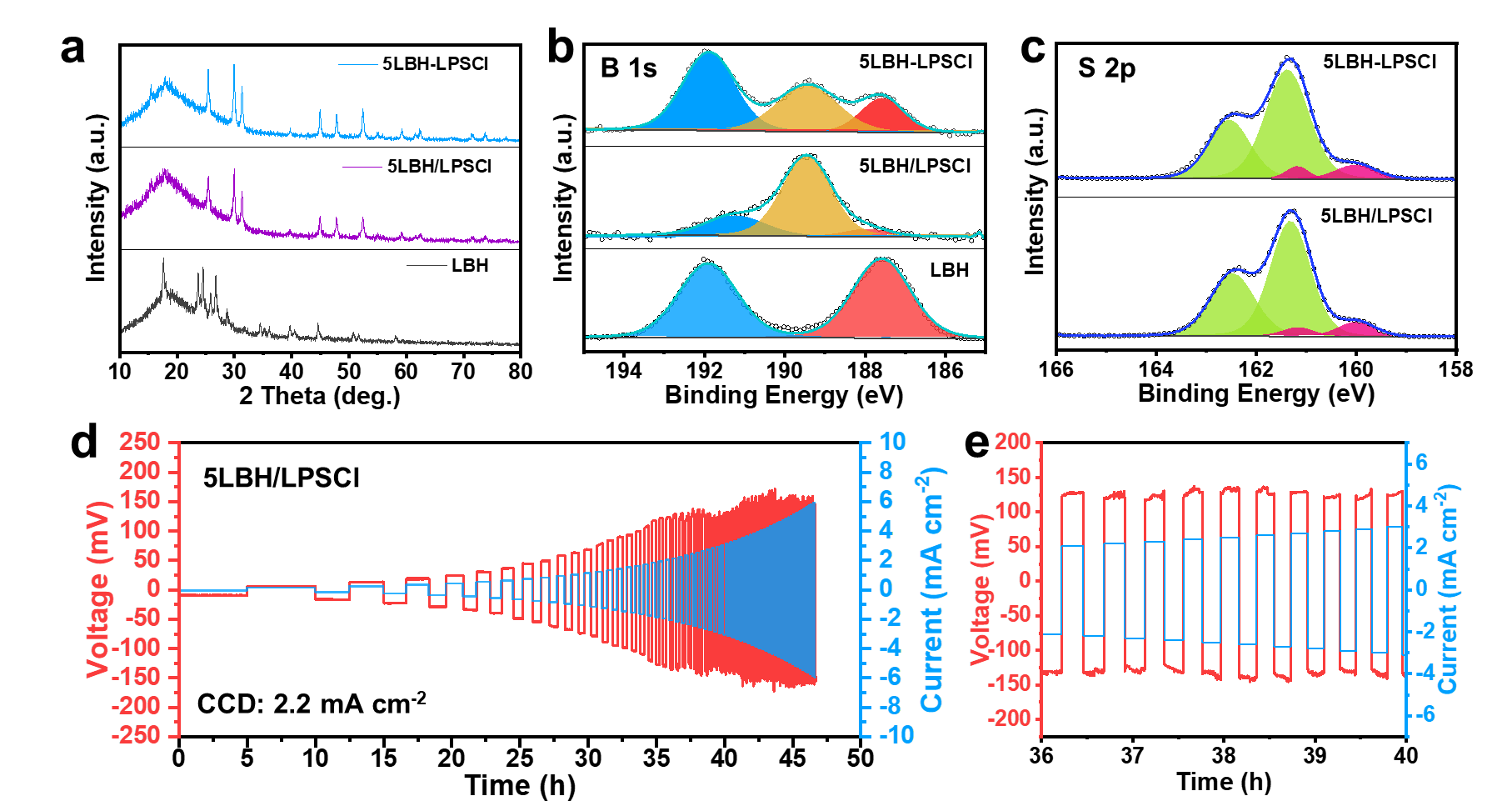


**Figure S21. (a)** XRD profiles, high resolution XPS **(b)** B 1s and **(c)** S 2p spectra of 5LBH-LPSCl, 5LBH/LPSCl, and LBH. **(d, e)** CCD test with representative galvanostatic profile of Li|5LBH/LPSCl|Li cell.


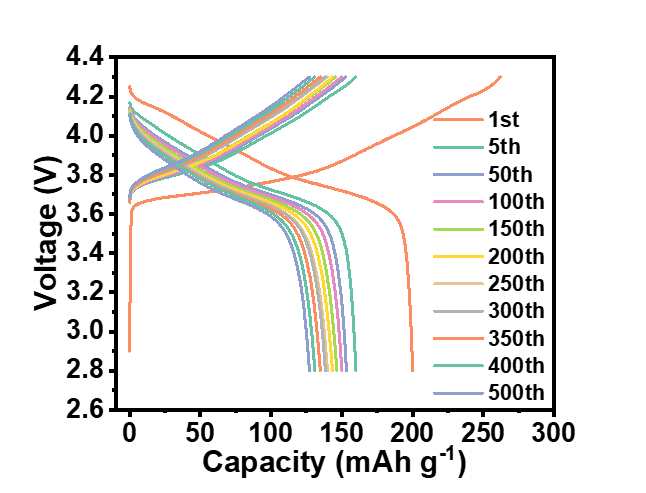


**Figure S22.** Galvanostatic profiles in selected cycles of Mg/W-Cu|5LBH-LPSCl|NMC AF-ASSB.


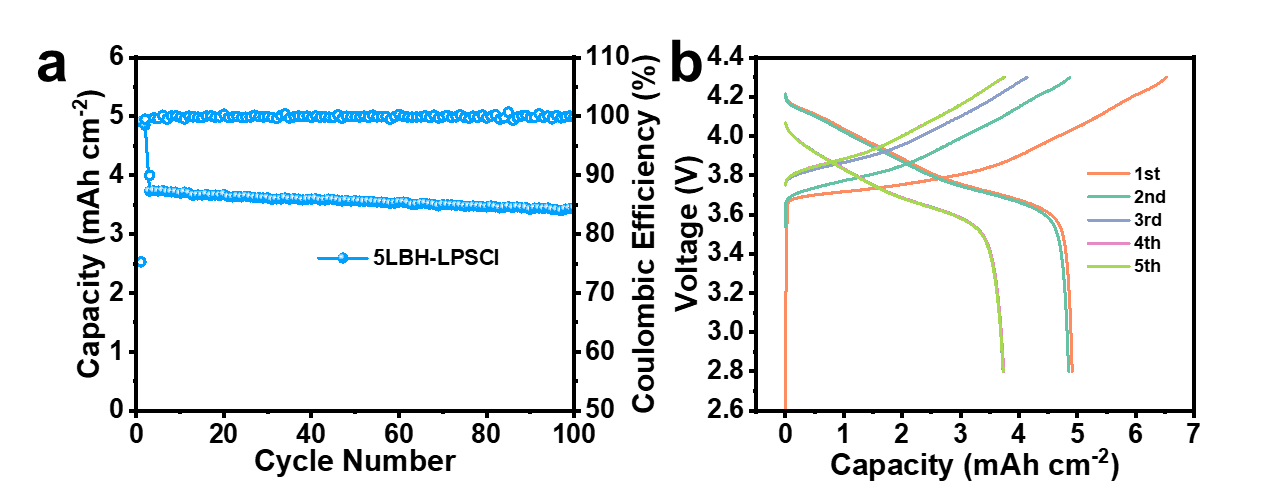


**Figure S23. (a)** Cycling performance of Mg/W-Cu|5LBH-LPSCl|NMC AF-ASSB with high NMC mass-loading, tested at 0.33C after initial 2 cycles at 0.1C. **(b)** Galvanostatic profiles of Mg/W-Cu|5LBH-LPSCl|NMC AF-ASSB during initial 5 cycles.


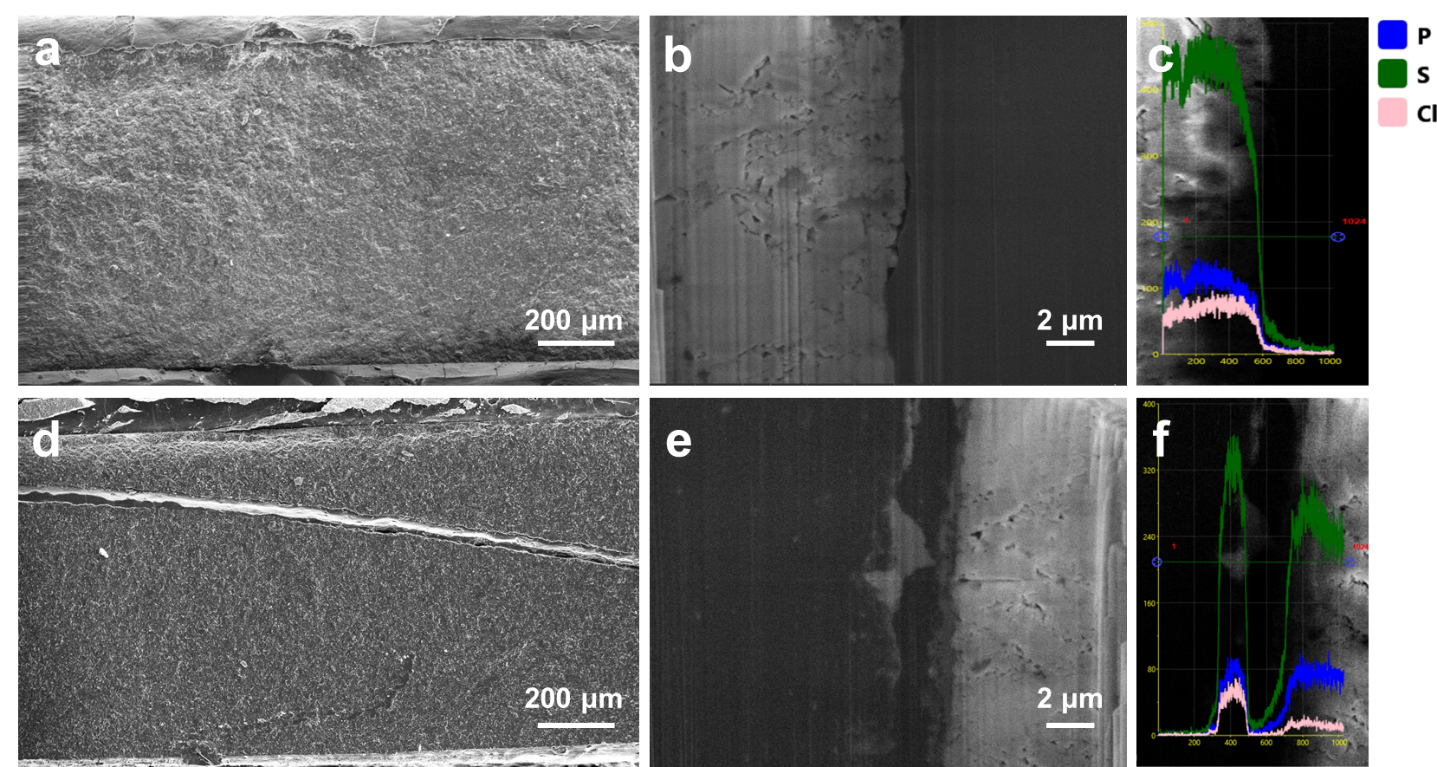


**Figure S24.** Fractured surface of the symmetric **(a)** Li|5LBH-LPSCl|Li and **(d)** Li|baseline-LPSCl|Li cells after 100 cycles at 1 mA cm^-2^ and 1 mAh cm^-2^. Cryo-FIB SEM cross-sectional images and associated EDXS maps of **(b, c)** Li|5LBH-LPSCl|Li and **(e, f)** Li|baseline-LPSCl|Li cells after 100 cycles at 1 mA cm^-2^ and 1 mAh cm^-2^.


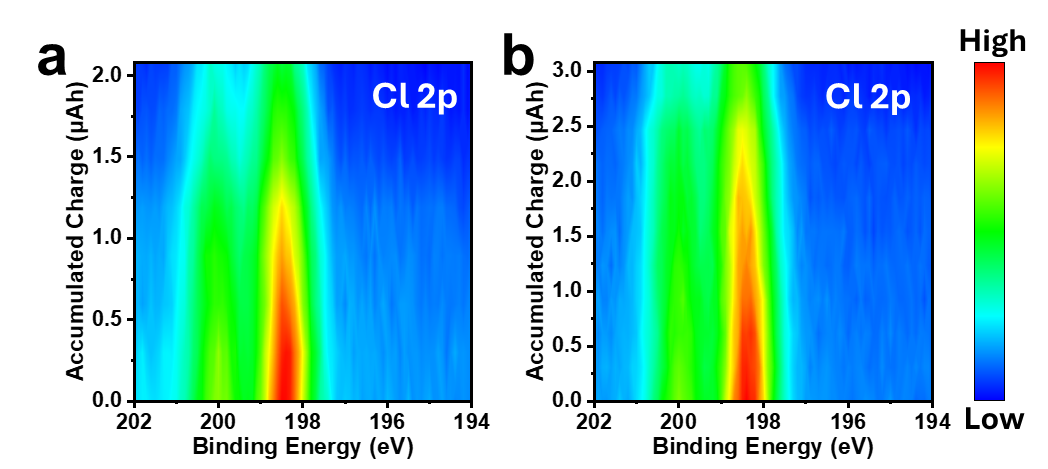


**Figure S25.** XPS contour plots of Cl 2p spectra for **(a)** 5LBH-LPSCl and **(b)** baseline-LPSCl SSEs.


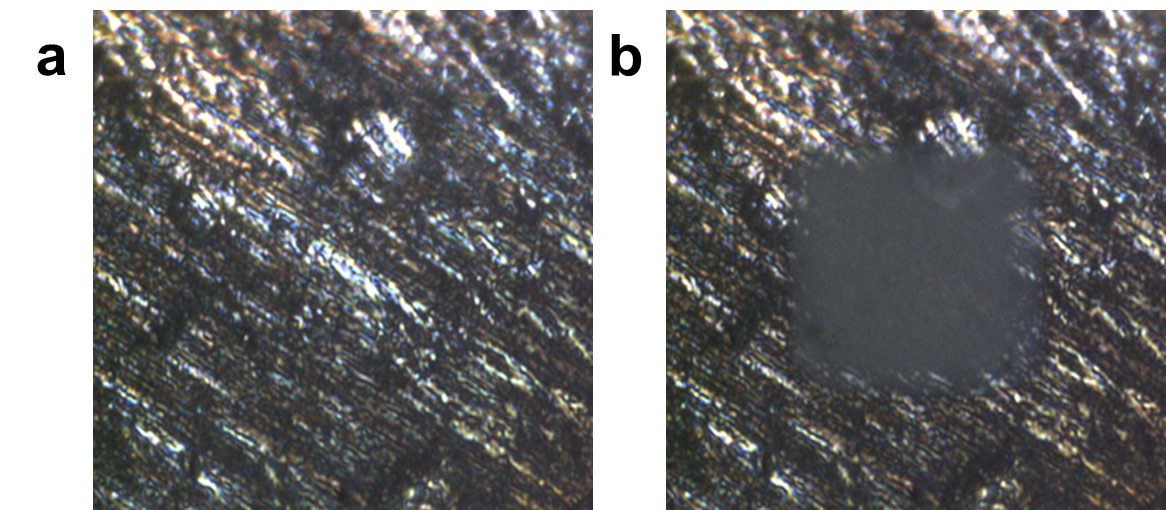


**Figure S26.** Optical images of the analyzed region for TOF-SIMs depth-profiling **(a)** before and **(b)** after sputtering.

**Table S1.** Reaction pathways and reaction energies of LPSCl with various organic Li salts predicted by Materials Project.

| **Reactants** | **Reaction Pathways** | **Reaction Energy (eV/atom)** |
| --- | --- | --- |
| LPSCl + LiBH_4_ | N/A | Does not react |
| LPSCl + LiBF_4_ | 0.65 Li_6_PS_5_Cl + 0.35 LiBF_4_ → 0.05 Li_5_B_7_S_13_ + 0.65 Li_3_PS_4_ + 0.65 LiCl + 1.4 LiF | -0.099 |
| LPSCl + LiPF_6_ | 0.5 Li_6_PS_5_Cl + 0.5 LiPF_6_ → 0.5 LiCl + 0.5 P_2_S_5_ + 3 LiF | -0.209 |
|  |  |  |
| LPSCl + LiDFOB | 0.375 Li_6_PS_5_Cl + 0.625 LiBC_2_(O_2_F)_2_ → 0.0625 Li_2_B_3_PO_8_ + 0.0625 Li_3_B_7_O_12_ + 0.375 LiCl + 0.3125 Li_3_PO_4_ + 0.1339 CS_14_ + 1.25 LiF + 1.116 C | -0.263 |
| LPSCl + LiTFSI | 0.5556 Li_6_PS_5_Cl + 0.4444 LiC_2_S_2_N(O_2_F_3_)_2_ → 0.5556 LiPO_3_ + 0.2619 CS_14_ + 0.05556 CO_2_ + 0.5556 LiCl + 2.667 LiF + 0.2222 N_2_ + 0.5714 C | -0.508 |

**Table S2.** Summary of the impedance data measured in **Figure S16**.

| Sample | 5LBH-LPSCl | | baseline-LPSCl | |
| --- | --- | --- | --- | --- |
| Cycle No. | R_b_/Ohms | R_SEI_+R_ct_ | R_b_ | R_SEI_+R_ct_ |
| 0 | 77.6 | 2.9 | 79.4 | 7.4 |
| 3 | 72.2 | 5.3 | 71.7 | 7.9 |
| 4 | 71.4 | 5.0 | 37.6 | 2.1 |
| 5 | 68.4 | 4.9 | 22.1 | 0.6 |
| 6 | 68.2 | 5.4 | 21.6 | 0.8 |

**Table S3.** Performance comparison of modified LPSCl SSE in the literature and this work.

| **Solid Electrolyte** | **CCD**  **mA cm^-2^** | **Cycling Performance** | **Reference** |
| --- | --- | --- | --- |
| **5LBH-LPSCl** | **7.3/CC***  **3.0/CT**** | **1 mA cm^-2^/1 mAh cm^-2^ for 1200 hours**  **2 mA cm^-2^/1 mAh cm^-2^ for 700 hours** | **This work** |
| Li_5.25_PS_4.25_(BH_4_)_1.75_ | 2.5/CT | 1 mA cm^-2^/1 mAh cm^-2^ for 1000 hours | ^[1]^ |
| Bi&O-doped LPSCl | 1.1/CT | 0.1 mA cm^-2^/0.1 mAh cm^-2^ for 600 hours | ^[2]^ |
| Sb&O-doped LPSCl | 1.5/CT | 0.1 mA cm^-2^/0.1 mAh cm^-2^ for 750 hours | ^[3]^ |
| In&O-doped LPSCl | 1.4/CT | 1 mA cm^-2^/1 mAh cm^-2^ for 400 hours | ^[4]^ |
| LiTFSI@LPSCl | 1.6/CT | 0.2 mA cm^-2^/0.2 mAh cm^-2^ for 1500 hours | ^[5]^ |
| Sn&O-doped LPSCl | 1.2/CC | 0.5 mA cm^-2^/0.5 mAh cm^-2^ for 400 hours | ^[6]^ |
| Densified LPSCl | 1.05/CT | 0.5 mA cm^-2^/0.5 mAh cm^-2^ for 3000 hours | ^[7]^ |
| ZnO doped LPSCl | 0.55/CT | 0.4 mA cm^-2^/0.2 mAh cm^-2^ for 100 hours | ^[8]^ |
| Sb-doped LPSCl | 1.2/CT | 0.1 mA cm^-2^/0.1 mAh cm^-2^ for 800 hours | ^[9]^ |
| Si-doped LPSCl | 1.9/CT | 1 mA cm^-2^/1 mAh cm^-2^ for 1000 hours | ^[10]^ |
| MgF_2_-doped LPSCl | 1.4/CT | 1 mA cm^-2^/1 mAh cm^-2^ for 200 hours | ^[11]^ |
| I-doped LPSCl | 1.65/CT | 0.1 mA cm^-2^/0.1 mAh cm^-2^ for 1200 hours | ^[12]^ |

*Constant capacity

**Constant time

**Table S4.** Performance comparison of reported NMC-based AF-ASSBs in the literature and this work.

| **Anode Materials** | **Solid Electrolyte** | **Mass Loading** | **Temperature** | **Capacity Retention** | **Reference** |
| --- | --- | --- | --- | --- | --- |
| **Mg/W-Cu** | **5LBH-LPSCl** | **1.5 mAh cm^-2^** | **RT** | **79% after 600 cycles at 0.33C** | **This work** |
|  |  | **6.5 mAh cm^-2^** |  | **92% after 100 cycles at 0.33C** |  |
| Mg/W-Cu | LPSCl | 1 mAh cm^-2^ | RT | 76% after 150 cycles at 0.33C | ^[13]^ |
| Cu_2_Te coated Cu | LPSCl | 6-12 mg cm^-2^ | RT | 80% after 50 cycles at 0.2C | ^[14]^ |
| Carbon felt & stainless steel | LPSCl | 10-20 mg cm^-2^ | RT | 55% after 100 cycles at 0.2C | ^[15]^ |
| Mxene/Mg | LPSCl | 20 mg cm^-2^ | RT | ~60 after 30 cycles at 0.1C | ^[16]^ |
| Surface etched stainless steel | LPSCl | ~16 mg cm^-2^ | 60 ^o^C | 74% after 5 cycles at 0.1C | ^[17]^ |
| Ag/C/stainless steel foil | LPSCl | ~16 mg cm^-2^ | 60 ^o^C | ~44% after 11 cycles at 0.1C | ^[18]^ |
| AgF/stainless steel | LPSCl | 50 mg cm^-2^ | RT | 85.4% after 50 cycles at 0.1C | ^[19]^ |
| Cu-Sn NTs@Cu | LPSCl | 2.9 mAh cm^-2^ | RT | 83.8% after 150 cycles at 0.1C | ^[20]^ |
| Ag-In coated stainless steel | LPSCl | 1.45 mAh cm^-2^ | 50 ^o^C | 80.2% after 250 cycles at 1C | ^[21]^ |
| Ag+ZnO coated stainless steel | LPSCl | 25 mg cm^-2^ | RT | 80.8% after 100 cycles at 1C | ^[22]^ |
| Cu@Ag | LPSCl+  polymer | - | RT | ~30% after 40 cycles at 0.1 mA cm^-2^ | ^[23]^ |

**Reference**

[1] J. H. Han, Y. Shin, Y. J. Lee, S. Ahn, Y. S. Lee, K. W. Yi, Y. W. Cho, *Small Methods* **2024**, 9, e2401046.

[2] H. Liu, Q. Zhu, C. Wang, G. Wang, Y. Liang, D. Li, L. Gao, L. Z. Fan, *Adv. Funct. Mater.* **2022**, 32, 2203858.

[3] C. Wei, C. Yu, R. Wang, L. Peng, S. Chen, X. Miao, S. Cheng, J. Xie, *J. Power Sources* **2023**, 559, 232659.

[4] C. Wang, J. Hao, J. Wu, H. Shi, L. Fan, J. Wang, Z. Wang, Z. Wang, L. Yang, Y. Gao, X. Yan, Y. Gu, *Adv. Funct. Mater.* **2024**, 34, 2313308.

[5] S. Jing, K. Wang, S. Li, Y. Lu, Y. Chen, K. Zhang, F. Li, S. Yin, Z. Zhang, F. Liu, *Energy Storage Mater.* **2025**, 76, 104131.

[6] G. Li, S. Wu, H. Zheng, Y. Yang, J. Cai, H. Zhu, X. Huang, H. Liu, H. Duan, *Adv. Funct. Mater.* **2022**, 33, 2211805.

[7] G. Liu, W. Weng, Z. Zhang, L. Wu, J. Yang, X. Yao, *Nano Lett.* **2020**, 20, 6660.

[8] G.-J. Jang, R. Rajagopal, S. Kang, K.-S. Ryu, *J. Alloys Compd.* **2023**, 957, 170273.

[9] H. Liu, Q. Zhu, Y. Liang, C. Wang, D. Li, X. Zhao, L. Gao, L.-Z. Fan, *Chem. Eng. J.* **2023**, 462, 142183.

[10] R. Song, J. Yao, R. Xu, Z. Li, X. Yan, C. Yu, Z. Huang, L. Zhang, *Adv. Energy Mater.* **2023**, 13, 2203631.

[11] C. Liu, B. Chen, T. Zhang, J. Zhang, R. Wang, J. Zheng, Q. Mao, X. Liu, *Angew. Chem. Int. Ed.* **2023**, 62, e202302655.

[12] R. Guo, Y. Zhong, P. Yu, K. Kang, S. Li, Z. Hu, X. Wang, C. Wu, Y. Bai, *Small* **2025**, 21, e2500764.

[13] Y. Wang, V. Raj, K. G. Naik, B. S. Vishnugopi, J. Cho, M. Nguyen, E. A. Recker, Y. Su, H. Celio, A. Dolocan, Z. A. Page, J. Watt, G. Henkelman, Q. H. Tu, P. P. Mukherjee, D. Mitlin, *Adv. Mater.* **2025**, 37, e2410948.

[14] Y. Wang, Y. Liu, M. Nguyen, J. Cho, N. Katyal, B. S. Vishnugopi, H. Hao, R. Fang, N. Wu, P. Liu, P. P. Mukherjee, J. Nanda, G. Henkelman, J. Watt, D. Mitlin, *Adv. Mater.* **2023**, 35, e2206762.

[15] D. Cao, T. Ji, Z. Wei, W. Liang, R. Bai, K. S. Burch, M. Geiwitz, H. Zhu, *Nano Lett.* **2023**, 23, 9392.

[16] J. Oh, S. H. Choi, J. Y. Kim, J. Lee, T. Lee, N. Lee, T. Lee, Y. Sohn, W. J. Chung, K. Y. Bae, S. Son, J. W. Choi, *Adv. Energy Mater.* **2023**, 13, 2301508.

[17] D. Gu, H. Kim, J.-H. Lee, S. Park, *J. Energy Chem.* **2022**, 70, 248.

[18] D. Gu, H. Kim, B.-K. Kim, J.-H. Lee, S. Park, *CrystEngComm* **2023**, 25, 4182.

[19] J. Lee, S. H. Choi, G. Im, K. J. Lee, T. Lee, J. Oh, N. Lee, H. Kim, Y. Kim, S. Lee, J. W. Choi, *Adv. Mater.* **2022**, 34, e2203580.

[20] J. Kim, S. Lee, J. Kim, J. Park, H. Lee, J. Kwon, S. Sun, J. Choi, U. Paik, T. Song, *Carbon Energy* **2024**, 6, e610.

[21] J. H. Lee, S.-H. Oh, H. Yim, H.-J. Lee, E. Kwon, S. Yu, J. S. Kim, J. Song, J. Koo, J. Cho, S. H. Kim, A. Ryu, S. H. Choi, Y. Kim, G. Im, J.-W. Choi, S.-H. Yu, *Energy Storage Mater.* **2024**, 69, 103398.

[22] Y. Sohn, J. Oh, J. Lee, H. Kim, I. Hwang, G. Noh, T. Lee, J. Y. Kim, K. Y. Bae, T. Lee, N. Lee, W. J. Chung, J. W. Choi, *Adv. Mater.* **2024**, 36, e2407443.

[23] N. T. Temesgen, H. K. Bezabh, M. A. Weret, K. N. Shitaw, Y. Nikodimos, B. W. Taklu, K. Lakshmanan, S.-C. Yang, S.-K. Jiang, C.-J. Huang, S.-H. Wu, W.-N. Su, B. J. Hwang, *J. Power Sources* **2023**, 556, 232462.
